# Single-cell lineage capture across genomic modalities with CellTag-multi reveals fate-specific gene regulatory changes

**DOI:** 10.1038/s41587-023-01931-4

**Published:** 2023-09-25

**Authors:** Kunal Jindal, Mohd Tayyab Adil, Naoto Yamaguchi, Xue Yang, Helen C. Wang, Kenji Kamimoto, Guillermo C. Rivera-Gonzalez, Samantha A. Morris

**Affiliations:** 1 Department of Developmental Biology, Washington University School of Medicine, St. Louis, MO USA; 2 Department of Genetics, Washington University School of Medicine, St. Louis, MO USA; 3 Center of Regenerative Medicine, Washington University School of Medicine, St. Louis, MO USA; 4 Department of Pediatrics, Division of Hematology and Oncology, Washington University School of Medicine, St. Louis, MO USA

**Keywords:** Genetic techniques, Epigenetics

## Abstract

Complex gene regulatory mechanisms underlie differentiation and reprogramming. Contemporary single-cell lineage-tracing (scLT) methods use expressed, heritable DNA barcodes to combine cell lineage readout with single-cell transcriptomics. However, reliance on transcriptional profiling limits adaptation to other single-cell assays. With CellTag-multi, we present an approach that enables direct capture of heritable random barcodes expressed as polyadenylated transcripts, in both single-cell RNA sequencing and single-cell Assay for Transposase Accessible Chromatin using sequencing assays, allowing for independent clonal tracking of transcriptional and epigenomic cell states. We validate CellTag-multi to characterize progenitor cell lineage priming during mouse hematopoiesis. Additionally, in direct reprogramming of fibroblasts to endoderm progenitors, we identify core regulatory programs underlying on-target and off-target fates. Furthermore, we reveal the transcription factor Zfp281 as a regulator of reprogramming outcome, biasing cells toward an off-target mesenchymal fate. Our results establish CellTag-multi as a lineage-tracing method compatible with multiple single-cell modalities and demonstrate its utility in revealing fate-specifying gene regulatory changes across diverse paradigms of differentiation and reprogramming.

## Abstract (web summary)

Lineage tracing using both transcriptomics and chromatin accessibility provides mechanistic insights into cell fate.

## Main

The quantification of cell identity is crucial to understanding development, disease and homeostasis, yet the notion of cell identity remains poorly defined^1^. Single-cell technologies, now tailored to diverse modalities^2^, are expanding our understanding of how cell identity is established and maintained^3^. In particular, single-cell lineage-tracing (scLT) methods allow cell relationships to be tracked throughout biological processes, revealing cell fate decisions during differentiation and reprogramming^4,5^. Prospective scLT methods label cells with unique genetic ‘barcodes’ that are expressed as RNA; capturing these barcodes through single-cell RNA sequencing (scRNA-seq) allows the parallel capture of lineage information and single-cell transcriptomes^6–13^.

**Fig. 1:**
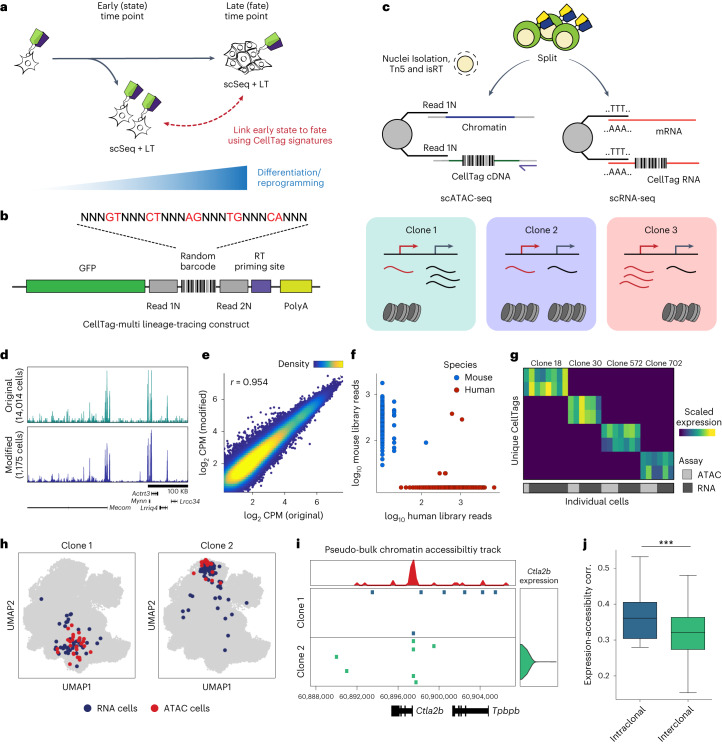
CellTag-multi allows simultaneous capture of lineage information with gene expression and chromatin accessibility. **a**, A framework for relating early cell state with fate using single-cell lineage tracing. **b**, Schematic depicting the CellTag-multi lineage-tracing construct. **c**, Schematic detailing parallel capture of CellTags during scRNA-seq and modified scATAC-seq library preparation, using targeted isRT of CellTags in intact nuclei. CellTag-multi enables simultaneous clonal tracking of transcriptional and epigenomic states. **d**, Browser tracks comparing chromatin accessibility signal across aggregated scATAC-seq profiles generated using the original and modified library preparation methods. **e**, Scatterplot comparing log-normalized reads in ATAC peaks across aggregated scATAC-seq profiles generated with the original and modified library preparation methods. *r* = Pearson correlation coefficient. **f**, Plot for the human–mouse species-mixing experiment depicting the number of CellTag reads per cell from each CellTag library (1,778 human cells and 275 mouse cells shown). **g**, Heatmap showing scaled CellTag expression in scRNA-seq and scATAC-seq siblings for four multi-omic clones identified in a population of expanded reprogramming fibroblasts. **h**, Joint UMAP of RNA and ATAC cells with cells from two clones (clone 1, 70 cells; clone 2, 119 cells) highlighted, along with assay information. **i**, Browser track showing single-cell accessibility at the *Ctla2b* locus and *Ctla2b* gene expression across clones 1 and 2. Top, pseudo-bulk accessibility signal at the *Ctla2b* locus. **j**, Boxplots comparing intraclonal and interclonal correlation between clonally aggregated gene expression and gene activity scores in the reprogramming dataset (*n* = 29 clones used; Mann–Whitney–Wilcoxon test, two-sided; ***P = 5.16 × 10^−^^4^). Boxplots: center line, median; box limits, first and third quartiles; whiskers, 1.5× interquartile range.

These methods to barcode and track cells have been deployed across several in vitro differentiation and reprogramming paradigms^4,5^. The accessibility of cells within these systems permits longitudinal sampling and cellular barcoding at precise time points, allowing early progenitor state to be linked to terminal fate (termed ‘state–fate analysis’; Fig. 1a). Such a strategy has been used to determine how well gene expression state in progenitors reflects eventual cell fate in hematopoiesis^12^. This work demonstrated that subsequent fate could not be predicted from progenitor gene expression alone, likely due to the existence of nontranscriptional, heritable determinants of cell fate, in addition to technical limitations of scRNA-seq. Similarly, viral barcoding, ‘CellTagging’, of transcription factor (TF)-mediated direct reprogramming of mouse embryonic fibroblasts (MEFs) to induced endoderm progenitors (iEPs), suggested that reprogramming outcome is determined during the early stages of fate conversion^7^. However, the early gene regulatory changes that set cells on their destined path have not been fully characterized. Additional information from epigenomic assays such as single-cell assay for transposase accessible chromatin by sequencing (scATAC-seq) may be crucial to uncover the heritable properties that have a key role in the establishment and maintenance of cell identity. Previously, natural DNA variation has been used to infer coarse cellular phylogenies with scATAC-seq^14,15^. However, the resolution of such retrospective methods is limited due to their reliance on the accrual of somatic mutations. In contrast, the density of lineage information recorded can be precisely controlled at biologically relevant time points using successive rounds of cellular barcoding^7,16^ with prospective methods. This is essential for profiling early, lineage-specific responses in dynamic systems such as differentiation and reprogramming.

To enable prospective lineage tracing with chromatin accessibility capture, we have developed ‘CellTag-multi’. CellTag-multi is based on our previous CellTagging technology, which uses sequential lentiviral delivery of CellTags (heritable random barcodes) to enable the construction of multilevel lineage trees^7,16^. Here we introduce a strategy in which CellTags, expressed as polyadenylated transcripts, can be captured in both scRNA-seq and scATAC-seq assays allowing for independent tracking of clonal transcriptional and epigenomic state.

We validate this method using in vitro hematopoiesis, a well-characterized model of multilineage differentiation, and demonstrate highly accurate reconstruction of lineage relationships and capture of lineage-specific progenitor cell states across scRNA-seq and scATAC-seq. Moreover, the addition of chromatin accessibility information to gene expression allows for an improvement in the prediction of differentiation outcome from early progenitor state. We also deploy CellTag-multi in the direct lineage reprogramming of fibroblasts to iEPs, to characterize early gene regulatory changes in rare subpopulations of cells that successfully reprogram. This application reveals how chromatin is remodeled following the expression of reprogramming TFs, enabling deeper insight into gene regulatory network reconfiguration. We uncover the TF Foxd2 as a facilitator of on-target reprogramming, increasing the efficiency of MEF to iEP conversion. Conversely, we identify Zfp281 as a TF biasing cells toward an off-target mesenchymal fate via its regulation of transforming growth factor-β (TGF-β) signaling, which we validate experimentally. We demonstrate that the identification of these TFs as reprogramming regulators is only possible via multi-omic profiling. Together, these findings highlight the utility of CellTag-multi in defining the molecular regulation of early cell state and its relation to fate across diverse biological applications.

## Development and validation of CellTag-multi

**Extended Data Fig. 1:**
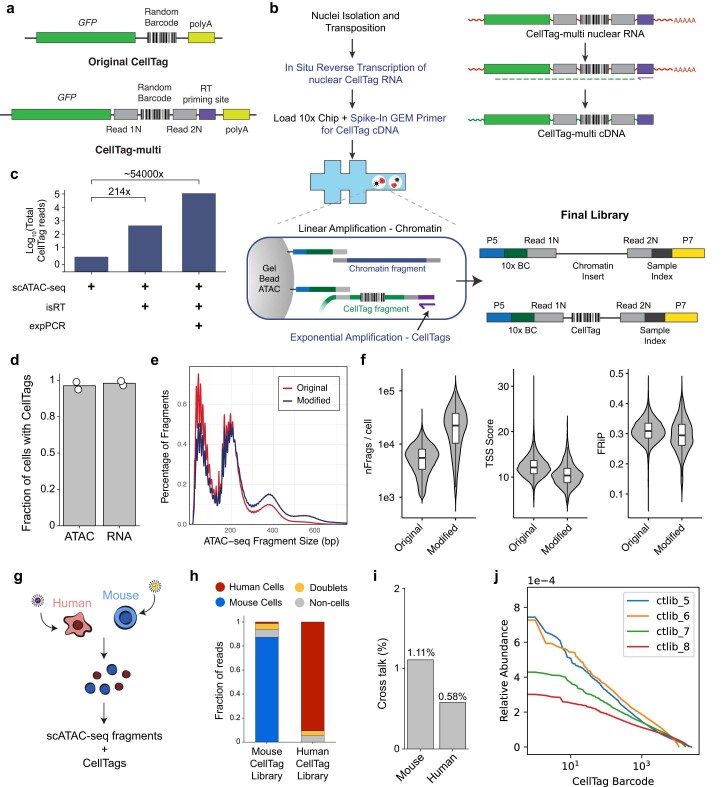
Development of CellTag-multi for parallel capture of lineage with scRNA-seq and scATAC-seq. (**a**) Schematic comparing the original^7,16^ CellTag lineage tracing construct to the CellTag-multi construct. (**b**) Left Panel: Detailed flow chart and schematic of the modified scATAC-seq library preparation protocol. Right Panel: Major molecular steps of the protocol and the final library containing both CellTag and chromatin accessibility fragments. (**c**) Bar plot comparing total number of CellTag reads per library obtained across different scATAC-seq library preparation methods. Each library was sequenced to a similar sequencing depth. (**d**) Mean percent cells with at least one CellTag detected in scATAC-seq, relative to scRNA-seq (n=2 samples/assay). Plots for (**e**) fragment size distribution and (**f**) various scATAC-seq quality metrics across two datasets generated using the ‘original’ and ‘modified’ scATAC-seq library preparation method (nFrags/cell: number of unique fragments per cell; FRiP: Fraction of reads in Peaks; Cell numbers – Original: 1000; Modified: 977). Boxplots: center line, median; box limits, first and third quartiles; whiskers, 1.5x interquartile range. (**g**) Schematic of the species mixing experiment to assess purity of CellTag signatures in scATAC-seq. (**h**) Bar plot depicting distribution of CellTag reads across, human, mouse, doublet and non-cell droplets for the two CellTag libraries. We observed that the majority of CellTag reads mapped to the expected species of origin, 87.2% for the mouse library and 91.4% for the human library. (**i**) Bar plot showing cross-talk levels (**Methods**) across the human and mouse cells profiled. (**j**) Line plots showing relative abundance of individual CellTag barcode across the four plasmid library preparations. The four individual libraries were pooled to obtain the final high complexity CellTag-multi library.

CellTagging relies on single-cell capture of CellTags—heritable DNA barcodes expressed as polyadenylated transcripts^7,16,17^. In the standard workflow, CellTags are captured as transcripts and reverse transcribed (RT), along with cellular mRNA, during 3′ end scRNA-seq library preparation. In contrast, scATAC-seq directly captures fragments of the accessible genome, omitting capture of CellTag transcripts, rendering CellTagging incompatible with scATAC-seq assays. To enable CellTag profiling with scATAC-seq, we introduced two essential modifications. First, we developed an in situ reverse transcription (isRT) step to selectively reverse transcribe CellTag barcodes inside intact nuclei. By introducing this additional step after transposition, we omitted the need to RT CellTags during scATAC-seq library construction. Second, we modified the CellTag construct to flank the random barcode with Nextera Read 1 and Read 2 adapters (Fig. 1b and Extended Data Fig. 1a,b).

During scATAC-seq library preparation, nuclei are partitioned into nanoliter droplets along with single-cell barcoding beads and PCR reagents. Each bead contains a barcoded forward primer complementary to the Nextera Read 1 adapter to barcode and linearly amplify all ATAC fragments during the gel bead-in-emulsion (GEM) incubation step. By inserting Nextera Read 1 and Read 2 adapters in the CellTag construct, we enabled single-cell capture of RT CellTags along with accessible chromatin during the GEM incubation stage (Fig. 1c and Extended Data Fig. 1b). This strategy improved the CellTag capture rate by >200-fold compared to the unmodified scATAC-seq protocol (Extended Data Fig. 1c). Additionally, we introduced a reverse primer specific to the CellTag cDNA during GEM incubation to exponentially amplify CellTag fragments, while ATAC fragments undergo linear amplification (Supplementary Table 1 and Extended Data Fig. 1b). Together, these modifications led to a >50,000-fold increase in CellTag capture (Extended Data Fig. 1c), with CellTags being detected in >96% of cells in scATAC-seq relative to 98% in scRNA-seq (Extended Data Fig. 1d), without negatively impacting scATAC-seq data quality or genome-wide chromatin accessibility signal (Fig. 1d,e and Extended Data Fig. 1e,f).

To support the accurate identification of clonally related cells, it is essential that CellTag signatures from individual cells are captured with high fidelity, minimizing background noise. To assess the fidelity of CellTag signatures captured in scATAC-seq, we performed a species-mixing experiment (Extended Data Fig. 1g). We labeled human (HEK 293T) cells and mouse (expanded iEPs) cells with two different versions of the CellTag-multi library, combined nuclei isolated from both populations in a 1:1 ratio and profiled them using our modified scATAC-seq method. Plotting CellTag reads per cell, we observed that nuclei from each species predominantly consisted of reads from the expected CellTag library, indicating minimal interspecies cross-talk (Fig. 1f and Extended Data Fig. 1h,i).

**Extended Data Fig. 2:**
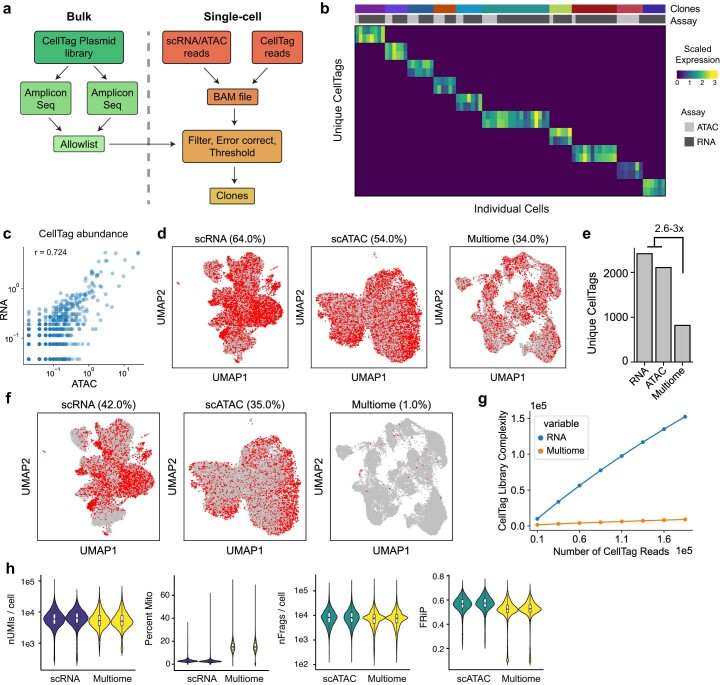
Testing CellTag-multi in cell lines and reprogramming fibroblasts. (**a**) Schematic depicting the workflow for CellTag library allow listing and clone identification from single-cell CellTag reads. (**b**) Heatmap depicting scaled CellTag expression across ten clones in a population of expanded reprogramming fibroblasts. (**c**) Correlation between CellTag abundance across scRNA-seq and scATAC-seq cells from the reprogramming dataset (Pearson’s correlation coefficient = 0.724). (**d**) UMAPs for CellTagged, expanded, reprogrammed fibroblasts profiled with scRNA-seq, scATAC-seq and 10x Multiome with cells containing any CellTag reads highlighted. The percentage of cells with any detectable CellTag reads in each dataset are mentioned above respective UMAPs. (21,637 scRNA-seq, 20,466 scATAC-seq and 20,231 Multiome cells shown) (**e**) Bar plot showing a reduction in the total number of unique tags detected after quality filtering in the Multiome cells as compared to scRNA and scATAC cells. (**f**) UMAPs comparing number of cells with CellTags after error correction, allow listing and filtering (**g**) Line plot comparing library complexity for CellTag amplicon libraries across scRNA and Multiome datasets at different read depths (**Methods**). scATAC cells are excluded from this comparison as those do not contain any UMIs. (**h**) Violin plots depicting key scRNA-seq and scATAC-seq quality metrics for the single modality and multiome assays.

Finally, to perform large-scale lineage-tracing experiments, we synthesized a complex CellTag-multi library containing ~80,000 unique barcodes, as confirmed by sequencing (Extended Data Fig. 1j and Supplementary Table 2; Supplementary Methods). We have also implemented a function to calculate the expected rate of homoplasy, the expected fraction of nonunique CellTag signatures in the starting cell population after CellTagging, in a simulated lineage-tracing experiment (Supplementary Methods). We applied CellTag-multi to a population of expanded mouse fibroblasts undergoing reprogramming to iEPs and profiled clones with scRNA-seq and scATAC-seq, detecting CellTags in 70% (RNA) and 51% (ATAC) of the cells at an average multiplicity of infection (MOI) of 2 (RNA) and 2.5 (ATAC). Filtering, error correction and allowlisting of CellTag reads (Methods) enabled high-fidelity identification of distinct clones across the two single-cell modalities (Fig. 1g,h and Extended Data Fig. 2a–c). As expected, the correlation between gene expression and accessibility was higher within clones than across clones (Fig. 1i,j). CellTag-multi enables multi-omic lineage tracing by independently profiling CellTags in scRNA and scATAC assays. An alternative to this approach would be co-assaying RNA and ATAC modalities from the same cell and retrieving CellTag reads from the gene expression data. However, when applied to a population of CellTagged cells, we observed a substantial decrease in number of cells with any CellTag reads, a 2.6–2.9-fold reduction in the number of unique CellTags detected, and a loss of CellTag amplicon unique molecular index (UMI) complexity with the 10X Genomics Multiome (RNA + ATAC) kit, as compared to scRNA-seq and scATAC-seq (Extended Data Fig. 2d–h) likely due to lower sensitivity of the multiome assay. These analyses establish the efficacy of CellTag-multi for the labeling and capture of clonally related cells across scRNA and scATAC modalities. Next, we leveraged CellTag-multi to link early state with cell fate in diverse cell fate specification and reprogramming paradigms.

## Benchmarking CellTag-multi using in vitro hematopoiesis

To validate lineage analysis across single-cell modalities with CellTag-multi, we applied it to hematopoiesis, a well-characterized paradigm for multilineage differentiation. Recently, scLT was used to define the early transcriptional cell states that lead to defined differentiation outcomes in mouse hematopoiesis. However, these analyses suggested that early transcriptional changes alone cannot fully define future cell fate and posited a role for cell states that evade transcriptional profiling, collectively termed hidden state variables^12^. In this context, we aimed to apply CellTag-multi to further refine state–fate linkages in early hematopoiesis by identifying fate-specific changes in both early gene expression and chromatin accessibility.

**Fig. 2:**
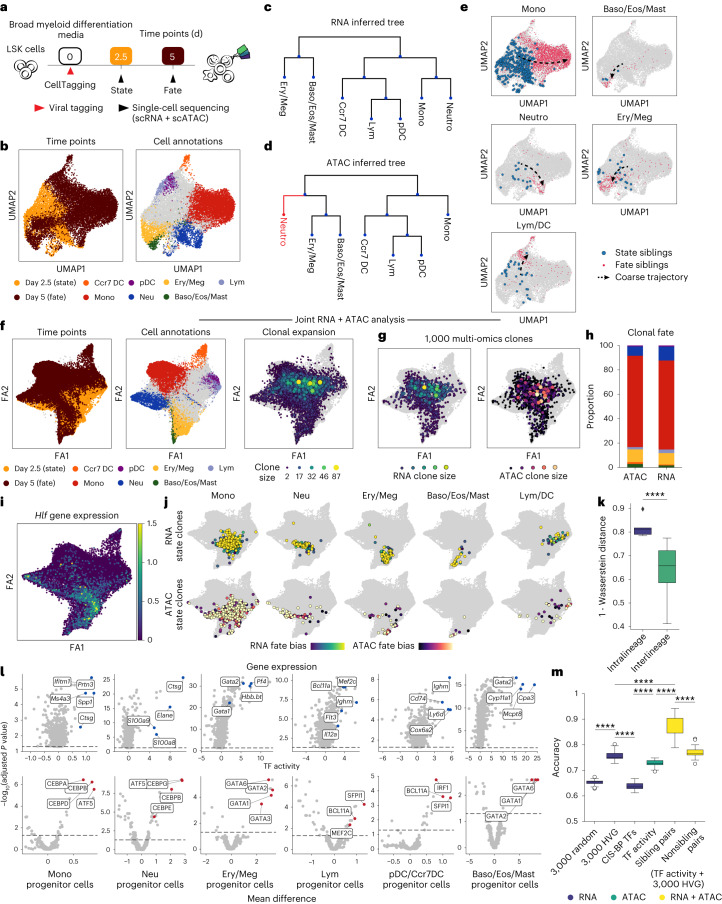
Application of CellTag-multi to link early hematopoietic cell state with fate. **a**, Schematic detailing the experimental design for the in vitro hematopoiesis state–fate experiment. **b**, scATAC-seq UMAPs with time point (left) and fate information (right) projected (Baso, Eos, Ery, Lym, Mast, Meg, Mono, Neu and pDC). Only major cell fates are highlighted. **c**,**d**, Hematopoietic fate hierarchy inferred from (**c**) scRNA or (**d**) scATAC clone coupling. **e**, scATAC-seq UMAPs with all state and fate siblings highlighted by fate. **f**, Clone-cell ForceAtlas (FA) embeddings with time point and fate projected onto cells (left and center) and clonal expansion information projected onto clones (right). **g**, FA embeddings with RNA and ATAC clonal expansion projected onto 1,000 multi-omic clones. Both modalities display expansion of early myeloid cells, consistent with our culture conditions. **h**, Bar plot of cell fates distribution across RNA and ATAC clones (fates colored as Fig. 2b). **i**, FA embedding with *Hlf* gene expression, a marker of hematopoietic stem and progenitor cells, projected onto the scRNA cells. **j**, FA embeddings with state (day 2.5) subclones highlighted for each major lineage along the differentiation continuum for both modalities and fate bias projected. **k**, Box plot comparing overlap between RNA and ATAC state subclones within and across cell fates (Mann–Whitney–Wilcoxon test, two-sided; *P* = 3.76 × 10^−^^5^; 5 intralineage and 20 interlineage comparisons). **l**, Volcano plots of differential feature enrichment analysis for each group of state subclones in scRNA (top) and scATAC (bottom). **m**, Box plot summarizing prediction accuracy values of trained state–fate prediction models. (Mann–Whitney–Wilcoxon test, two-sided; ****P < 0.0001, highly variable genes (HVG), *n* = 25 accuracy values for each model (Methods)). Boxplots, center line and median; box limits, first and third quartiles; whiskers, 1.5× interquartile range. Baso, basophils; Eos, eosinophils; Ery, erythroids; Lym, lymphoids; Mast, mast cells; Meg, megakaryocytes; Mono, monocytes; Neu, neutrophils; pDC: plasmacytoid dendritic cells.

**Extended Data Fig. 3:**
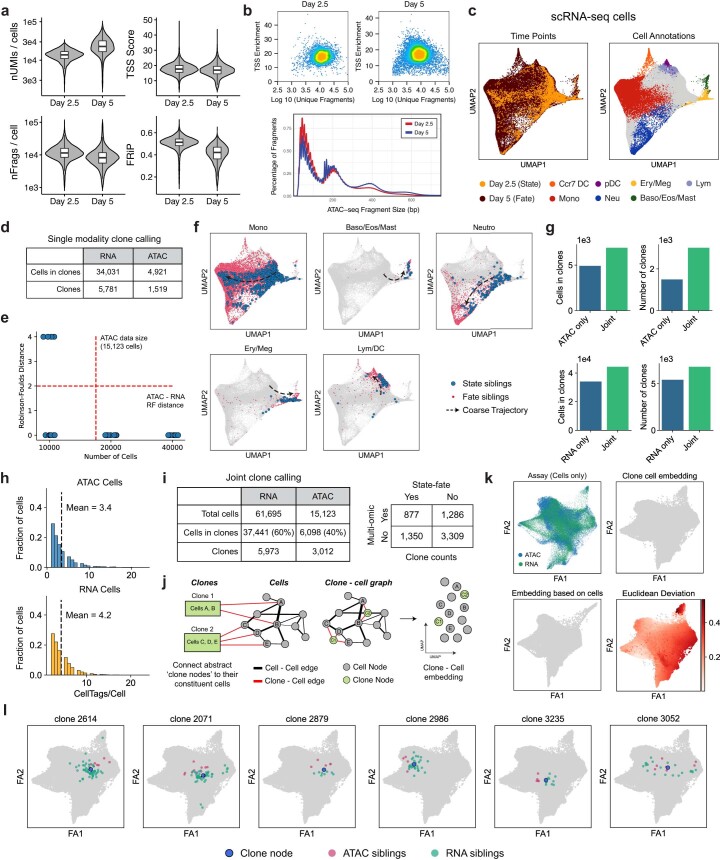
Single-cell metrics and cell annotation in hematopoiesis. (**a**) Violin plots for single-cell quality metrics for the scRNA-seq and scATAC-seq data. Day 2.5: 5,161 (RNA) and 4,628 (ATAC) cells; Day 5: 56,534 (RNA) and 10,495 (ATAC) cells. (**b**) Unique fragments/cell vs single-cell TSS enrichment scatterplots and fragment size distribution plot for the two scATAC-seq time-points. (**c**) scRNA-seq UMAPs with time point (left panel) and cell fate information (right panel) projected. (**d**) Table summarizing clones identified in scRNA and scATAC datasets independently. (**e**) RNA fate hierarchy trees built with fewer cells are more discordant with the tree built using the full dataset (n=10 rounds of subsamplings for each indicated cell count). (**f**) scRNA-seq UMAPs with state and fate siblings for major hematopoietic fates highlighted. (**g**) Bar plots comparing number of clones and cells in clones across single-modality and joint modality clone calling. (**h**) Histograms of CellTags detected per cell across scRNA-seq and scATAC-seq datasets after filtering and processing of CellTag reads. (**i**) Tables summarizing all clones identified in the dataset. (**j**) Workflow for joint embedding of cells and clone nodes. (**k**) Top Left: Clone-cell embedding with RNA and ATAC assay information projected (cells only). Top Right and Bottom Left: Comparison of cell embeddings obtained using a conventional FA embedding vs a joint clone-cell graph-based embedding (only cell nodes shown, for direct comparison). Bottom Right: Clone-cell graph FA embedding with cells colored by deviation in their position between the two embeddings. (**l**) Visualization of clones along with their constituent cells confirms that clone nodes faithfully represent cells. Boxplots in a: center line: median; box limits: first and third quartiles; whiskers: up to 1.5x interquartile range.

We isolated Lin^−^, Sca1^+^, c-Kit^+^ (LSK) cells from adult mouse bone marrow and cultured them in broad myeloid differentiation media^12^. Upon isolation, we tagged these cells with the CellTag-multi library to track clones across modalities. To capture both early state and fate across clones, we profiled half of the cells 60 h after initiation of differentiation (day 2.5; state sample), replated the remaining cells across two technical replicates and collected them for sequencing on day 5 (fate sample). In the case of both samples, cells were split between scRNA-seq and scATAC-seq (Fig. 2a), resulting in the profiling of 9,789 state cells (scRNA-seq, *n* = 5,161; scATAC-seq, *n* = 4,628) and 67,029 fate cells (scRNA-seq, *n* = 56,534; scATAC-seq, *n* = 10,495 cells), after quality filtering (Extended Data Fig. 3a,b). We identified cells from all major hematopoietic lineages across single-cell modalities (Fig. 2b and Extended Data Fig. 3c). CellTagging was consistent across single-cell modalities, yielding 83–99% labeled cells (expected rate of homoplasy = 0.0036).

To compare clonal analysis across modalities, we first analyzed the scRNA-seq and scATAC-seq datasets separately and identified clones in each modality independently (Extended Data Fig. 3d). Fate hierarchies inferred using clonally related cells (Methods) were highly consistent across scRNA and scATAC (Fig. 2c,d; Robinson–Foulds (RF) distance = 2; Methods), with only the neutrophils being misplaced in the hierarchy inferred from scATAC data. This discrepancy may have arisen due to the smaller size of the scATAC dataset (Extended Data Fig. 3e). Assigning a fate label to each clone, based on the modal (most abundant) cell type among its day 5 siblings, allowed mapping of coarse fate trajectories on the 2D embeddings (Fig. 2e and Extended Data Fig. 3f). These analyses demonstrated the ability of CellTag-multi in defining fate relationships using clonal scATAC-seq data alone.

Joint clone calling across both datasets led to an increase in number of cells tracked (Extended Data Fig. 3g), likely due to clones that are split across modalities (multi-omic clones). We identified a total of 37,441 scRNA-seq cells in 5,973 clones and 6,098 scATAC-seq cells in 3,012 clones, labeled with 4.2 CellTags/cell (in scRNA-seq) and 3.4 CellTags/cell (in scATAC-seq) on average (Extended Data Fig. 3h,i). In total, 2,227 clones spanned both state and fate samples, including 877 multi-omic clones. These clones were used for the remainder of the analyses.

**Extended Data Fig. 4:**
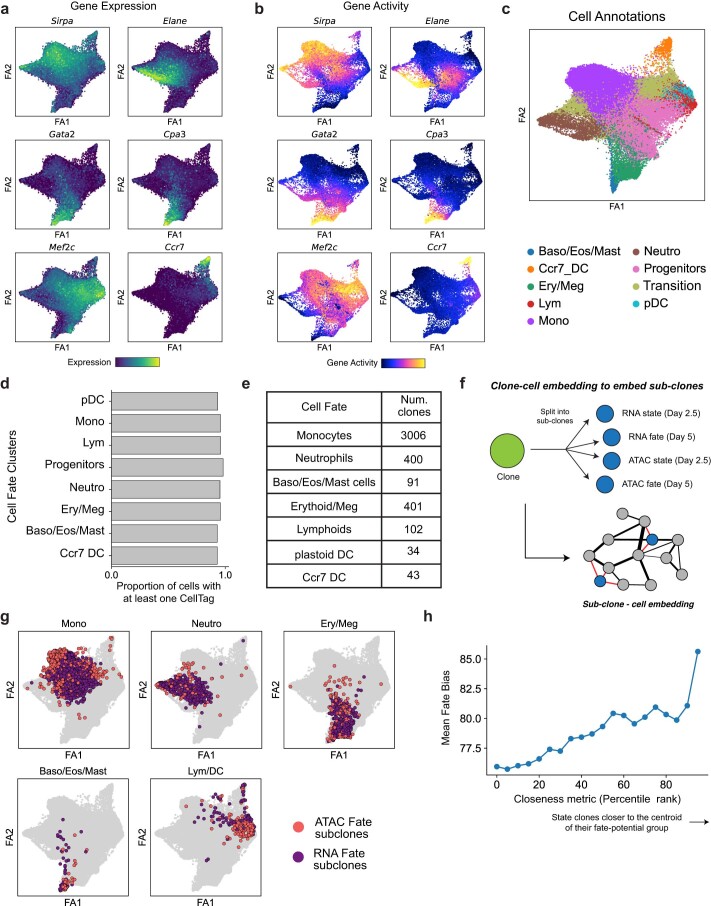
Fate annotation in hematopoiesis. (**a**) Marker gene expression and (**b**) accessibility projected on the FA embedding for various hematopoietic cell fates. (**c**) FA embedding with the full set of cell annotations in the hematopoiesis dataset projected. (**d**) Bar plot summarizing proportion of cells with at least one detectable CellTag across major cell fate clusters. CellTags are profiled uniformly across all cell states. (**e**) Table summarizing number of clones identified in each fate. Clonal fate was annotated using the most dominant cell type amongst Day 5 fate siblings. (**f**) Schematic depicting joint embedding of sub-clones with cells using the clone-cell embedding method. (**g**) FA embedding with fate sub-clone nodes for major lineages highlighted. (**h**) Plot showing that fate bias increases from the periphery of each state group towards the center. The closeness metric is directly proportional to the closeness of a state sub-clone node to the centroid of its state group in a 30-dimensional UMAP space (**Methods**).

For visualization, we co-embedded cells from both modalities using canonical correlation analysis (CCA), a data integration approach that works by identifying shared sources of variation across datasets^18^. Furthermore, we devised a unique clone-cell co-embedding approach to include clones as individual data points in a single-cell embedding, enabling straightforward visualization and assessment of clone-level metadata and global trends across clones (Extended Data Fig. 3j and Supplementary Methods). We first extracted the cell–cell similarity graph, produced as part of standard single-cell analysis workflows. In this graph, each cell is represented by a node and the connection between a pair of cells is weighted based on their phenotypic similarity. Next, we imputed abstract clone nodes and clone-cell edges to this graph based on clonal data. Finally, we used this expanded clone-cell graph as input for dimensionality reduction algorithms such as uniform manifold approximation and projection (UMAP)^19^ or ForceAtlas^20^ to produce a single 2D-embedding of the data, where both cells and clones are represented by individual points. We applied this visualization to the hematopoiesis data to co-embed RNA and ATAC cells with all clones, with minimal impact on the underlying structure of the data (Fig. 2f and Extended Data Fig. 3k). Clones, now represented as individual data points, faithfully represented their constituent cells (Extended Data Fig. 3l) and can be used to visualize clonal metadata across all cells (Fig. 2f, right). Consistent with previous reports, we observe continuous transitions from progenitor populations to distinct hematopoietic lineages across modalities, as previously reported^12,21,22^ (Extended Data Fig. 4a–c). While CellTag capture was uniform across cell states (Extended Data Fig. 4d), we observed higher clonal expansion along the monocyte lineage, consistent with our myeloid differentiation culture conditions (Fig. 2f (right), 2g).

We linked day 2.5 cell state with day 5 fate, by re-assigning each clone, from the joint clone calling results, a fate label based on the modal cell type among its day 5 siblings (Fig. 2h and Extended Data Fig. 4e). To map early clonal state along the differentiation continuum, we extended our clone-cell embedding approach further and split each clone into subclones (up to four) based on the assay and time point capture of each sibling (Extended Data Fig. 4f). While day 5 fate subclones localized largely within their respective cell fate clusters (Extended Data Fig. 4g), day 2.5 state subclones associated with each major fate formed distinct groups closer to the undifferentiated progenitors (Fig. 2i,j), suggesting early functional priming of immature cells. Moreover, state subclones within the same ‘fate potential’ group overlapped significantly across single-cell modalities (Mann–Whitney–Wilcoxon test; *P* = 3.76 × 10^−5^; Fig. 2j,k), demonstrating high-fidelity capture of state–fate linkages across transcriptional and epigenomic states with CellTag-multi. Projecting fate bias scores, defined as the fraction of fate siblings belonging to the assigned clonal fate, onto state subclones, we observed that low fate bias clones occupied areas closer to the overlapping boundaries of each fate potential region, likely indicating areas of multipotency (Fig. 2j and Extended Data Fig. 4h).

To characterize these fate-specific changes in early cell state on a molecular level, we assessed the enrichment of transcriptional and epigenetic signatures in day 2.5 siblings for each fate group (Fig. 2l; Methods). With gene expression, we observed enrichment of several known fate-specific markers in each group, such as *Spp1* (ref. ^12^) and *Ms4a3* (ref. ^23^) in the monocyte-primed group; *Elane* and *Ctsg*^12^ in the neutrophil-primed group; *Pf4* (ref. ^24^) and *Gata2* (ref. ^12^) in the erythroid/megakaryocyte groups. In the lymphoid group, we identified *Flt3*, a predominantly lympho-myeloid gene^25^, and several lymphoid fate-specific genes such as *Mef2c*^26^ and *Bcl11a*^27^. For epigenetic data, we focused on TF activity scores, which estimate the enrichment of TF motifs in single-cell epigenomes^28^. Unlike peak accessibility, TF activity feature space is dense and continuous, allowing comparison between small groups of cells, and is easier to interpret relative to individual peak features. TF activity enrichment analysis revealed several expected lineage specifying TFs for each fate^21,29^, such as several C/EBP family TFs enriched in monocyte- and neutrophil-primed groups; GATA1 and GATA2 in the erythroid/megakaryocyte and basophils/eosinophils/mast cells groups; lympho-myeloid TF SFPI1 (also known as PU.1) in the lymphoid and dendritic cells (DCs) group, along with BCL-family and MEF2 TFs, indicating extensive epigenomic priming in early cells toward their respective cell fate. A complete list of differential gene expression and TF activity enrichment can be found in Supplementary Table 3. Gene Ontology (GO) analysis for marker genes for each group can be found in Supplementary Table 4.

## Chromatin accessibility and gene expression jointly define fate-predictive cell state

Our abovementioned state–fate analysis suggests that lineage-specific changes in gene expression are accompanied by extensive epigenetic remodeling, rendering the genome more accessible to fate-specifying TFs. Previous analysis has suggested that cell states hidden from transcriptional profiling have a role in fully defining fate-associated changes in cell state^12^. Changes in chromatin accessibility could account for some of this hidden variance, and we tested this hypothesis by assessing whether cell fate can be accurately predicted from an early state using our multi-omic clonal data.

**Extended Data Fig. 5:**
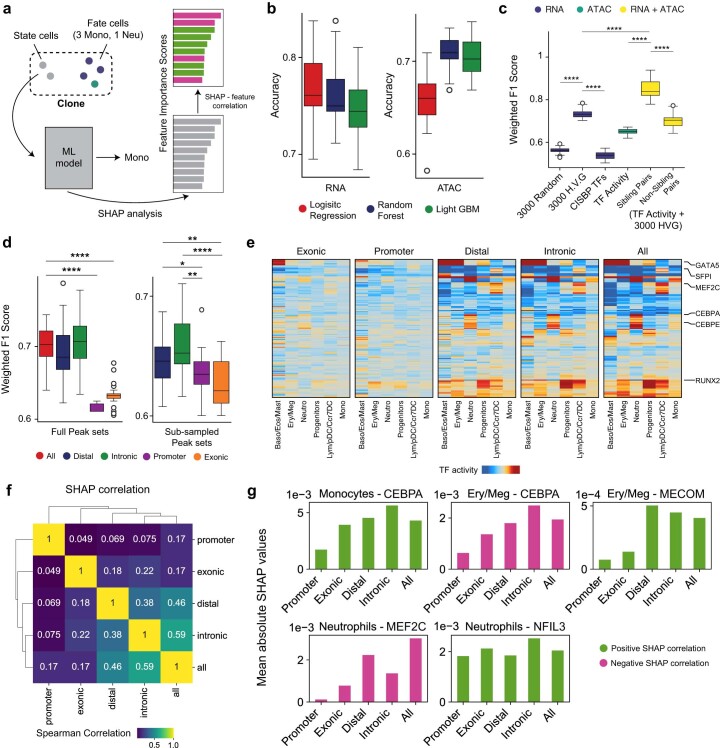
Machine learning analysis to predict cell fate from state. (**a**) Schematic of state-fate prediction analysis. (**b**) Accuracy values obtained with the three model architectures for either RNA (left) or ATAC (right) data (n=25 accuracy values/boxplot). (**c**) Same plot as Fig. 2m but for F1-weighted scores (Mann Whitney Wilcoxon test, two-sided, n=25 values/boxplot). (**d**) Boxplots showing variation in F1-weighted score values for ATAC models trained on full peak sets for ‘all’, ‘distal’, ‘intronic’, ‘exonic’ or ‘promoter’ peaks (left) and subsetted ‘distal’, ‘intronic’, ‘exonic’ and ‘promoter’ peak sets(right; n = 8823 peaks; Mann Whitney Wilcoxon test, two-sided; n=25 accuracy values/boxplot). (**e**) Heatmaps depicting mean TF activity scores for fate predictive TFs across groups of state siblings. TFs show strong fate biased enrichment patterns in ‘distal’, ‘intronic’ and ‘all’ peaks but not exonic and promoter datasets. (**f**) Heatmap depicting Rank correlation of SHAP values for top predictive TFs shows high similarity between ‘distal’, ‘intronic’ and ‘all’ peaks models. (**g**) Bar plots of mean absolute SHAP values for a few TFs for fates as indicated. Bars are colored based on value of SHAP correlation. SHAP analysis reveals that motif activity of many lineage specifying TFs is less predictive of cell fate in ‘promoter’ and ‘exonic’ models, while remains comparable across models for some others. Positive SHAP correlation for a feature in a given fate implies that higher values of the feature lead to higher probability of the model outputting that fate label. Negative correlation indicates lower values of the feature lead to higher probability of the model outputting that fate label. All boxplots: center line, median; box limits, first and third quartiles; whiskers, 1.5x interquartile range. For c and d: p-values: **** = p < 0.0001; ** = p < 0.01; * = p < 0.05. Exact p-values in Supplementary Table 12.

We trained machine-learning models to predict clonal cell fate from gene expression or chromatin accessibility profiles of day 2.5 siblings (Extended Data Fig. 5a). We tested the following three different architectures: logistic regression, random forest and LightGBM, and assessed model performance using prediction accuracy (Extended Data Fig. 5b). Overall, random forest models performed the best and were used for all downstream analysis. For gene expression, we trained a classification model to predict clonal fate using expression of the 3,000 most highly variable genes (HVG) and obtained an accuracy of 75.6% (Fig. 2m and Extended Data Fig. 5c). For chromatin accessibility, we used day 2.5 imputed TF activity scores (Methods) for 884 TF motifs to predict the clonal fate and obtained an accuracy of 72.7% (Fig. 2m). Notably, an RNA model trained on expression levels of TFs, obtained from the Catalog of Inferred Sequence Binding Preferences database, only scored only 63.8% on prediction accuracy (Fig. 2m). The significantly lower predictive performance of TF expression compared to TF activity could be attributed to either technical dropout in scRNA-seq or significantly higher lineage-specific priming of TF binding sites compared to TF expression, or a combination of both.

To assess fate-specific priming in different functional regions of the epigenome, we computed TF activity scores using subsets of accessible peaks and compared fate prediction performance across these feature spaces. Specifically, we computed TF activity scores using only promoter, distal, exonic or intronic peaks and trained fate prediction models with each. We observed significant variation in performance among different ATAC models, indicating different levels of fate-specific epigenetic priming across functional regions of the genome (Extended Data Fig. 5d). This variation was independent of the number of peaks used to compute each set of TF activity scores (Extended Data Fig. 5d). Distal and intronic were the best performing models, comparable in performance to the full peak set model (All). Promoter and exonic models performed significantly worse, suggesting that fate-specifying epigenetic changes during these early stages were dominated by changes in distal regulatory regions of the epigenome rather than the accessibility of genes themselves. This observation is reinforced by the persistence of TF enrichment trends across state groups in distal and intronic subsets but not in the exonic and promoter subsets (Extended Data Fig. 5e). We confirmed these results using SHapley Additive exPlanations (SHAP), a game theoretic approach to quantify the contributions of individual input features in explaining the output of a machine-learning model^30^. Indeed, SHAP analysis showed that in the better-performing models, an increase in CEBP/A motif accessibility and an increase in MECOM motif accessibility were better predictors of monocyte and Ery/Meg fates, respectively, suggesting a lack of functional priming in the promoter-proximal accessible genome (Extended Data Fig. 5f,g).

Finally, we tested whether combining RNA and ATAC features is more predictive of fate than either individual modality. For this, we trained a combined RNA and ATAC model where RNA and ATAC day 2.5 siblings within the same clone were paired randomly, and their combined gene expression and TF activity signatures were used to predict clonal fate label. This analysis was limited to multi-omic state–fate clones. The combination of both state modalities was significantly better at predicting fate (mean accuracy score = 86.5%) compared to either individual modality or pairs of unrelated RNA and ATAC state cells (Fig. 2m). These results show that both gene expression and chromatin accessibility jointly comprise cell states that define future cell fate. Moreover, these modalities consist of nonredundant and highly complementary state information, as a combination of both predicts cell fate much more accurately than each modality in isolation.

## Dissecting clonal dynamics of direct reprogramming

Our application of CellTag-multi to hematopoiesis demonstrated the method’s utility to capture informative gene regulatory dynamics in a well-characterized differentiation paradigm. We next applied CellTag-multi to a less defined system—the direct reprogramming of MEFs to iEPs driven by retroviral overexpression (OE) of *Hnf4α* and *Foxa1* (refs. ^7,31,32^). Direct lineage reprogramming presents a unique paradigm of cell identity conversion, with cells often transitioning through progenitor-like states or acquiring off-target identities^33,34^. Such nonlinear fate dynamics can be challenging to assess, especially when relying solely on the computational inference of cell fate trajectories^13^. Ground truth lineage tracing serves as a crucial resource for dissecting lineage-specific cell-state changes during direct reprogramming^7^. Originally reported to yield hepatocyte-like cells^31^, we have previously shown that Hnf4α and Foxa1 OE in MEFs generates cells with the broader potential to functionally engraft liver and intestine^17,32,35^. This prompted their redesignation as iEPs. More recently, we have further characterized the similarity of long-term cultured iEPs to regenerating biliary epithelial cells (BECs)^36^.

Using our original CellTag-based lineage tracing, we identified the following two distinct iEP reprogramming trajectories: a successful ‘reprogrammed’ trajectory, characterized by endodermal and hepatic gene expression, and a ‘dead-end’ trajectory, defined by a failure to extinguish the starting fibroblast identity^7^. Further work demonstrated key functional differences between these fates, with successfully reprogrammed cells harboring the potential to engraft acutely damaged mouse intestine^17^. Our previous lineage tracing suggests that the reprogrammed and dead-end fates are determined in the early stages of fate conversion^7^. However, our original CellTagging methodology did not capture any epigenetic information and only sparsely sampled early-state clones, limiting mechanistic insight into these initial reprogramming stages.

**Fig. 3:**
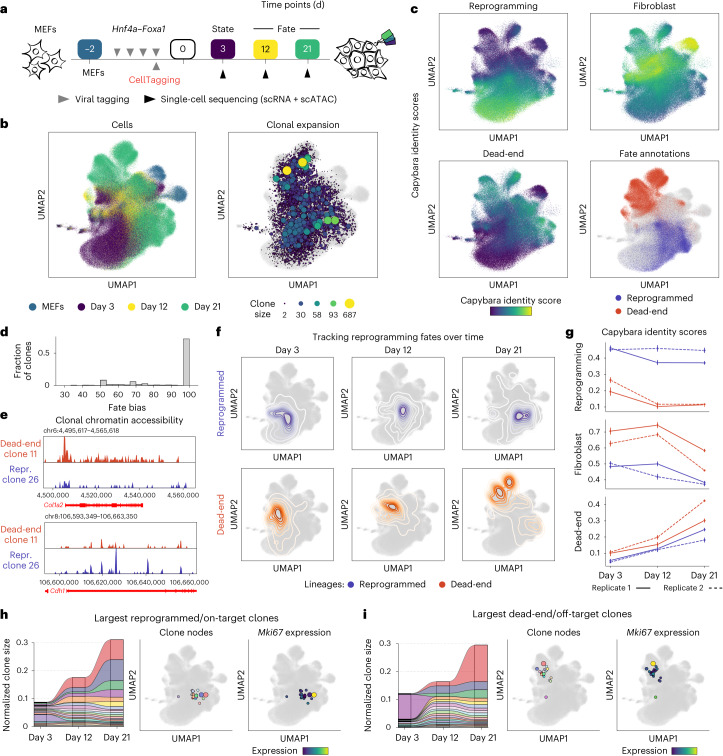
Application of CellTag-multi to dissect clonal fate dynamics in direct reprogramming. **a**, Experimental design for the direct reprogramming state–fate experiment. **b**, Cells from both scRNA-seq and scATAC-seq, across all time points, were co-embedded with clones and visualized using a UMAP. Left, time point information projected on cells. Right, clonal expansion visualized using clone nodes. **c**, Capybara transcriptional identity scores projected on scRNA-seq cells for reprogrammed, dead-end and fibroblast cell identities, based on a previous lineage-tracing dataset^7^. Cell fates were annotated for days 12 and 21. Reprogrammed and dead-end cell fates are highlighted (lower right). **d**, Histogram of fate bias scores across all state–fate clones. Fate bias scores were calculated using cells from days 12 and 21. **e**, Clonal chromatin accessibility browser tracks for one dead-end and one reprogramming clone. **f**, Contour plots showing longitudinal tracking of cell fates enabled by CellTag-multi. **g**, Transcriptional identity dynamics tracked along both lineages. Dead-end cells depart from a MEF-like identity and acquire an off-target reprogrammed state. **h**,**i**, Significant clonal expansion is observed along both lineages, as depicted via alluvial plots, clone nodes and clonal expression levels of *Mki67* (a proliferation marker gene) in the 20 largest (**h**) reprogramming/on-target and (**i**) dead-end/off-target clones.

**Extended Data Fig. 6:**
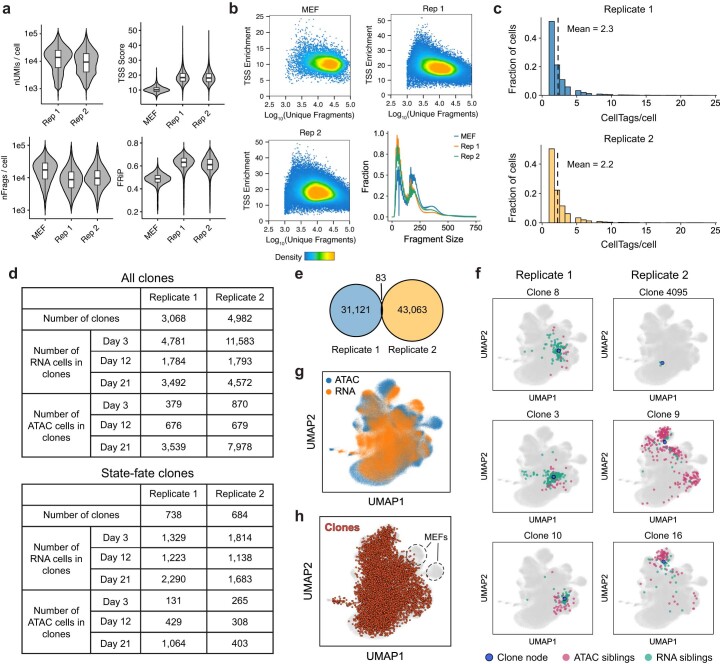
Single-cell metrics for the direct reprogramming dataset. (**a**) Single-cell quality metrics for the scRNA-seq and scATAC-seq datasets, split by biological replicates. Cell numbers - MEF: 10,119; Rep 1: 92,261 (RNA) and 92,367 (ATAC); Rep 2: 123,827 (RNA) and 121,200 (ATAC). (**b**) Unique fragments/cell vs single-cell TSS enrichment scatterplots and fragment size distribution plots for the scATAC-seq dataset. (**c**) Histograms of number of CellTags detected per cell across the two biological replicates after filtering and processing of CellTag reads. (**d**) Summary of all clones identified across single-cell modalities, for both biological replicates. (**e**) Venn diagram showing overlap of CellTag signatures across the two biological replicates. (**f**) UMAPs depicting representative clone nodes from both biological replicates along with their constituent cells. (**g**) Cells in the clone-cell embedding UMAP with assay information projected shows uniform embedding of both single-cell modalities. (**h**) UMAP with all clone nodes highlighted shows uniform distribution of clones across all cell states except the unlabeled MEFs.

Here we deployed CellTag-multi in iEP reprogramming, modifying our clonal resampling strategy to optimize state–fate analysis (Fig. 3a). First, we transduced MEFs with Hnf4α and Foxa1 for 48 h to initiate reprogramming, in two independent biological replicates. During the last 12 h of this 48-h period, we transduced cells with the complex CellTag-multi library, enabling clonal relationships to be tracked. Seventy-two hours following the final viral transduction (reprogramming day 3), we collected two-thirds of the cells for single-cell RNA and ATAC profiling (state sample) and replated the remaining cells. Subsequent samples were collected on days 12 and 21 (fate samples) to assess reprogramming outcome. We also profiled the starting MEF population (scATAC-seq, this study; scRNA-seq from a previous study^7^), resulting in a total of 450,300 single cells (scATAC-seq, 223,686; scRNA-seq, 226,614) in the final dataset after quality filtering (Extended Data Fig. 6c,d). We identified a total of 8,050 clones, containing 42,081 cells (replicate 1, 3,068 clones; replicate 2, 4,982 clones; average clone sizes of 4.8 and 5.5 cells per clone, respectively (Extended Data Fig. 6c–e); expected rate of homoplasy, 0.0053; observed rate of homoplasy, 0.001). We identified 1,422 ‘state–fate’ clones across both replicates. These clones were characterized as clones spanning the initial state (day 3) and at least one of the subsequent fate time points, either day 12 or day 21 (Extended Data Fig. 6d).

**Extended Data Fig. 7:**
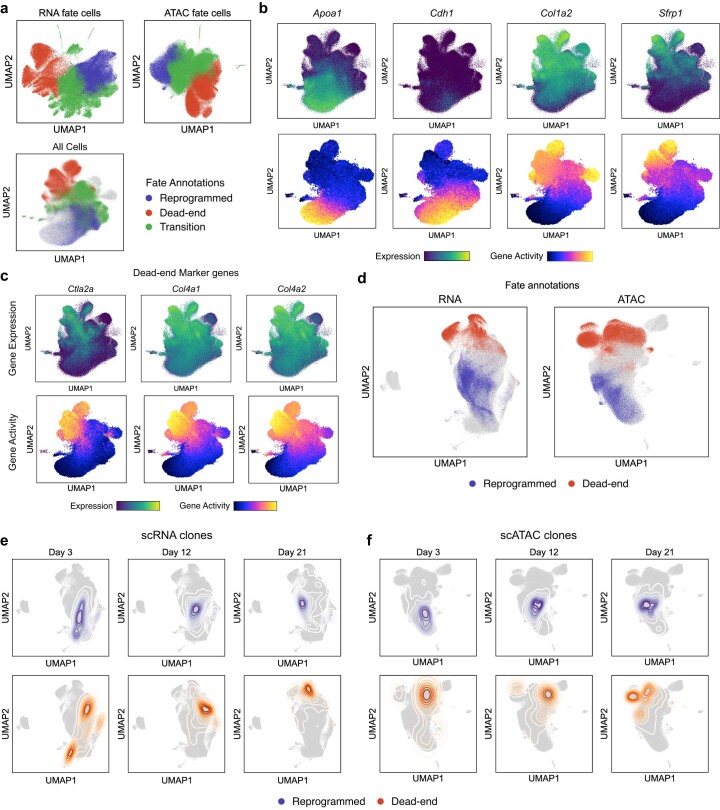
Fate annotation in direct reprogramming. (**a**) UMAPs with ‘reprogrammed’, ‘dead-end’ and ‘transition’ fate information projected. Fate cells (Days 12 and 21) were re-clustered and annotated with one of the three fates based on marker gene expression/accessibility, in both modalities independently. (**b**) Clone-cell embedding UMAPs with expression and accessibility information for key marker genes projected. (**c**) UMAPs with expression and accessibility information of key dead-end marker genes projected. (**d**) UMAPs for individual modalities with reprogrammed and dead-end fate information projected. (**e,f**) Contour plots showing longitudinal tracking of cell fates enabled by CellTag-multi, independently for both scRNA and scATAC.

Following dimensionality reduction and clustering of the co-embedded RNA and ATAC datasets, clone-cell embedding was performed (Fig. 3b and Extended Data Fig. 6f–h). We annotated days 12 and 21 fate clusters (‘reprogrammed’, ‘dead-end’ and ‘transition’) based on expression and accessibility of known reprogramming associated genes and unsupervised cell type classification based on transcriptional state using Capybara^36^ (Fig. 3c and Extended Data Fig. 7a,b). Capybara is a computational tool to score cell identities at single-cell resolution using quadratic programming. In line with our previous reports^7,17,36,37^, reprogrammed cells express epithelial and iEP markers, *Cdh1* and *Apoa1*, respectively. Dead-end cells are characterized by the retention of fibroblast gene expression but are still transcriptionally distinct from MEFs, expressing low levels of iEP markers and several dead-end-specific genes such as *Sfrp1*, a Wnt signaling modulator^7^ (Extended Data Fig. 7b,c). Transition cells represent states in between MEFs and reprogrammed/dead-end identities. Following cluster annotation, we assigned fate labels to each state–fate clone. As the majority of state–fate clones showed high fate bias, we assigned clonal fate based on the modal cell type among the fate siblings (Fig. 3d), identifying 1,018 reprogrammed, 2,024 dead-end and 1,395 transition clones. Dead-end and reprogrammed clones displayed a lineage-specific increase in accessibility of known marker genes (Fig. 3e).

Using clonal information, we linked each reprogrammed and dead-end clone to its day 3 state siblings, allowing us to track changes in cell identity longitudinally (Fig. 3f). These results were consistent when clonal analysis was performed for each modality independently (Extended Data Fig. 7d–f). Comparing Capybara transcriptional cell identity scores across lineages, we found that iEP identity scores were consistently higher along the reprogrammed lineage compared to the dead-end lineage. MEF identity scores, while significantly higher along the dead-end lineage, exhibited a steep decline after day 12 coinciding with an increase in dead-end transcriptional identity score (Fig. 3g). This suggested a delayed departure from MEF identity to an alternate cell state. We observed high levels of clonal expansion along both lineages (Fig. 3h,i). These observations suggest that despite retaining expression of canonical fibroblast marker genes, dead-end cells are in a fundamentally distinct, off-target cell state and reprogramming outcome. Thus, the ‘reprogrammed’ and ‘dead-end’ fates are better described as ‘on-target’ and ‘off-target’ reprogramming, respectively.

## State–fate linkage reveals off-target reprogramming features

**Extended Data Fig 8:**
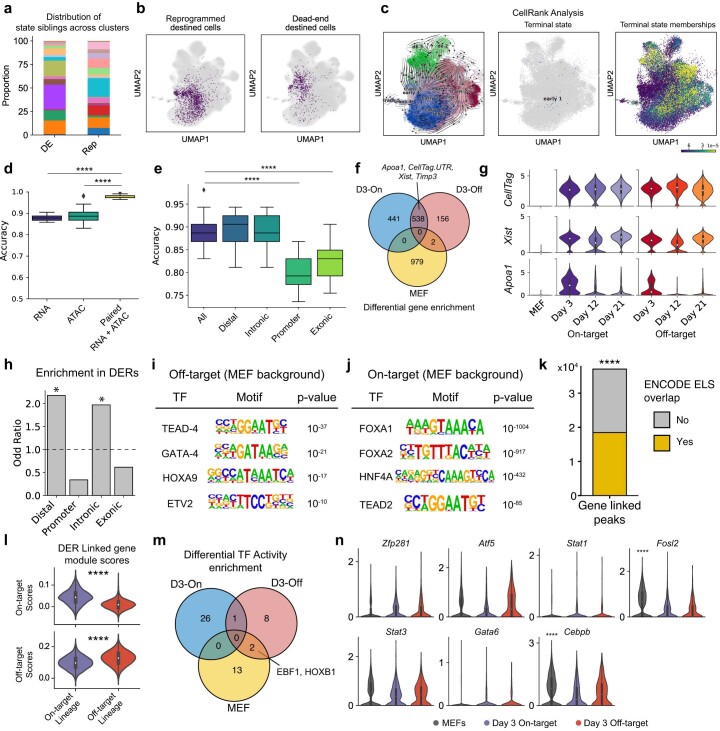
Differential analysis of expression and chromatin accessibility state across lineages. (**a**) Distribution of reprogramming and dead-end destined cells across clusters and (**b**) their projection on the clone-cell embedding UMAP. (**c**) CellRank fails to reveal true lineage dynamics underlying reprogramming. Velocity vectors overlaid onto the UMAP (left). ‘Early_1’, a cluster from Day 3 cells identified as a terminal state (middle). Continuous membership values for the terminal state ‘Early_1’ (right). (**d**) Fate prediction from Day 3 cell state using random forest classifiers. (Mann Whitney Wilcoxon test, two-sided; p-values: Paired vs ATAC = 3.5e-09; Paired vs RNA = 1.4e-09; n=25 accuracy values/boxplot). (**e**) State-fate prediction analysis using subsets of peaks (Mann Whitney Wilcoxon test, two-sided; p-values: All vs Promoter = 1.757e-08; All vs Exonic = 1.052e-07; n=25 accuracy values/boxplot). (**f**) Differentially enrichment genes across uninduced MEFs and the two fates on Day 3. (**g**) Violin plots for several genes enriched in both reprogramming fates on Day 3. (**h**) DERs are enriched in distal and intronic regions of the genome. (Fischer’s exact test, one-sided; p-values: 0 for both intronic and distal peaks). HOMER analysis to identify motifs enriched in (**i**) Off-target (dead-end) DERs and (**j**) On-target (reprogrammed) DERs, compared to a MEF DER background. (**k**) Enrichment of ENCODE cCRE Enhancer Like Elements in gene linked peaks. (Permutation test, one-sided; 10,000 permutations, p-value: 1e-04). (**l**) Enrichment of DER linked genes’ module scores in each lineage (Mann Whitney Wilcoxon test, two-sided; p-values: top = 6.2e-221; bottom = 0). (**m**) Differentially enriched TF activities across uninduced MEFs and the two reprogramming fates on Day 3. (**n**) Violin plots showing expression of off-target TFs, as identified from TF activity analysis, across uninduced MEFs and the two fates on Day 3. *Cdx1* expression was not detected in any of the groups and is hence not plotted (Bonferroni corrected p-values: *Cebpb* = 1.64e-14, *Fosl2 =* 1.37e-39). All boxplots: center line, median; box limits, first and third quartiles; whiskers, 1.5x interquartile range. Panels g, l and n: Cell numbers – MEF: 10,526; Others – as indicated in Extended Data Fig. 6d.

Next, to identify early state changes that regulate entry onto distinct fate trajectories, we focused on day 3 state clones destined to on-target (reprogrammed) or off-target (dead-end) reprogramming fates. From assessing the distribution of day 3 siblings destined to either of the two fates, it is evident that they are not localized to defined clusters (Extended Data Fig. 8a,b). Furthermore, trajectory inference using CellRank, an unsupervised trajectory inference method based on RNA velocity and Markov modeling^38^, fails to reveal these initial states (Extended Data Fig. 8c), demonstrating the importance of ground truth lineage tracing. We found that both day 3 gene expression and TF activities were highly predictive of clonal fate. Similar to our analysis of hematopoiesis, fate prediction accuracy was significantly higher when both modalities were considered, as compared to either modality individually. Furthermore, distal and intronic peaks were more predictive of fate than proximal and exonic (Extended Data Fig. 8d,e).

**Fig. 4:**
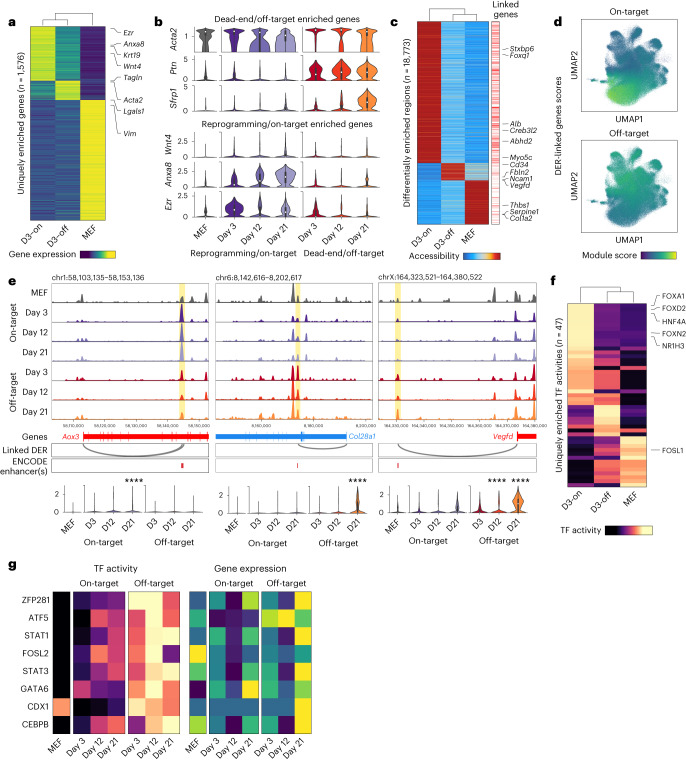
Assessing fate-specific changes in early cell state. **a**, Heatmap of genes uniquely enriched across uninduced MEFs or one of the two reprogramming fates on day 3 (false discovery rate (FDR) threshold = 0.05, log fold-change threshold = 0; D3-on: Day 3 on-target destined cells, D3-off: Day 3 off-target destined cells). **b**, Violin plots of several genes enriched in either off-target (dead-end) destined or on-target (reprogramming) destined cells. **c**, Heatmap of peaks uniquely enriched across uninduced MEFs or one of the two reprogramming fates on day 3 (FDR threshold = 0.05, log fold-change threshold = 1). Right, annotation of peaks linked to genes (Methods). **d**, Module scores for genes linked to either on-target or off-target DERs projected onto the clone-cell embedding. **e**, Top, accessibility browser tracks for each lineage split by day, highlighting peaks linked to late lineage markers (on-target: *Aox3*; off-target: *Col28a1* and *Vegfd*) showing lineage-specific changes in accessibility on day 3. The *Aox3*- and *Vegfd*-linked DERs overlap perfectly with an ENCODE Enhancer-Like Signature (ELS) element, while the *Col28a1*-linked DER is within 100 bp of an ELS. Bottom, expression levels of the three genes across MEFs and the two reprogramming lineages split by days (Mann–Whitney–Wilcoxon test; two-sided; Bonferroni corrected ****P < 0.0001). **f**, Heatmap of TF activities uniquely enriched across uninduced MEFs or one of the two reprogramming fates on day 3 (FDR threshold = 0.05, mean difference threshold = 0.5). **g**, Heatmap showing TF activity (left) and gene expression (right) levels for off-target associated TFs in MEFs and each reprogramming lineage split by time points. TF activity scores show a much stronger lineage bias as compared to gene expression. Box plot definitions for **b** and **e**—center point, median; box limits, first and third quartiles; whiskers, up to 1.5× interquartile range; cell numbers—as indicated in Extended Data Fig. 6d.

To identify early molecular signatures of lineage specification, we compared gene expression, chromatin accessibility and TF activity scores across MEFs and day 3 state siblings grouped by fate outcome. Comparing gene expression enrichment across the three groups, 2,116 genes were differentially enriched with 1,576 enriched genes uniquely defining each group (Fig. 4a and Extended Data Fig. 8f). While some genes displayed transient fate-specific expression, others consistently increased over time in a lineage-specific manner (Supplementary Table 5). Early iEP marker genes such as *Apoa1* were enriched in both on-target and off-target trajectories on day 3, consistent with our previous observation that most cells initiate reprogramming^7^ (Extended Data Fig. 8f,g). On-target (reprogrammed) enriched genes included *Krt19*, a marker of BECs, Wnt signaling associated genes *Wnt4*, *Anxa8* and epithelial marker *Ezr* (Fig. 4b and Supplementary Table 6). Top off-target (dead-end)-related genes included canonical smooth muscle markers *Acta2* and *Tagln* and other mesenchymal genes such as *Ptn* and *Ncam1*, suggesting broad engagement of mesenchymal programs, in addition to *Sfrp1*, a Wnt signaling pathway inhibitor (Fig. 4b and Supplementary Table 6).

Comparing genome-wide chromatin accessibility revealed 18,773 differentially enriched regions (DERs) across day 3 on-target and off-target destined cells and uninduced MEFs, indicating extensive fate-specific epigenetic reconfiguration during early reprogramming (Fig. 4c and Supplementary Table 7). DERs were enriched for distal and intronic peaks, suggesting epigenetic repatterning of distal regions as a driver of cell fate conversion, consistent with our above observations in hematopoiesis (Extended Data Fig. 8h). Motif analysis revealed enrichment of reprogramming and hepatic TFs in on-target DERs, and several TFs with documented roles in mesenchymal fates^39,40^ in off-target DERs (Extended Data Fig. 8i,j). Using our paired RNA and ATAC data, we linked accessible peaks to genes and identified 37,058 putative *cis*-regulatory elements (CREs)^41^ (Fig. 4c; Methods). Gene-linked peaks were enriched for Enhancer-like Signature (ELS) elements from the ENCODE candidate CRE database^42^ (Methods; Extended Data Fig. 8k). Genes linked to on-target and off-target DERs displayed fate-specific expression patterns (Fig. 4d and Extended Data Fig. 8l). On-target DERs consisted of several CREs linked to endodermal genes, such as *Alb*, *Foxq1* and *Creb3l2*. In contrast, off-target DERs contained CREs linked to mesenchymal genes such as *Ncam1*, a modulator of mesenchymal stromal cell migration^43^, *Fbln2*, a mesenchymal gene associated with embryonic heart development^44^ and *Vegfd*, a regulator of angiogenesis^45^ and endothelial differentiation of bone-marrow-derived mesenchymal stem cells^46^ (Fig. 4c and Supplementary Table 7). In several instances, this analysis captured lineage-specific changes in accessibility of CREs before significant changes in gene expression were detected. For instance, a *Vegfd*-linked CRE overlapping with an ENCODE enhancer displayed enrichment in dead-end destined cells (day 3), while expression changes were not detectable until day 12. Similar regulatory changes were observed for *Aox3* (ref. ^47^), a liver-associated aldehyde oxidase, and *Col28a1*, an oligodendrocyte enriched collagen^48^, before changes in gene expression (Fig. 4e and Supplementary Table 7).

To identify functional changes in chromatin accessibility on a genomic scale, we compared inferred TF activities across on-target and off-target destined cells and uninduced MEFs. To preclude potential false positives, we discarded all TFs with low correlation (<0.3) with their respective gene activity scores, identifying 47 uniquely enriched TFs (Fig. 4f, Extended Data Fig. 8m and Supplementary Table 8). On-target destined cells were highly enriched for the two reprogramming TFs, FOXA1 and HNF4A. Other on-target associated TFs included FOXD2 and NR1H3, a hepatic fate-specifying TF^49^ (Fig. 4f). We identified a set of eight TFs uniquely enriched in off-target destined cells (Fig. 4f,g). Several of these TFs (Zfp281, Cebpb and Gata6) have been previously documented to have a role in regulating mesenchymal cell identities^50–52^. Surveying the expression data, none of the off-target TFs display a similar fate-biased enrichment (Fig. 4g and Extended Data Fig. 8n), highlighting the importance of lineage-specific chromatin profiling in identifying these targets. This lack of enrichment could be due to technical dropout during scRNA-seq or due to secondary mechanisms regulating the genomic engagement of these TFs.

Altogether, our lineage-specific multi-omic assessment of iEP generation demonstrates clear early molecular differences associated with reprogramming outcomes. Indeed, from as early as reprogramming day 3, cells on the dead-end lineage exhibit unique characteristics. Rather than retaining MEF identity, we observe that the dead-end lineage constitutes a highly proliferative, mesenchymal cell state with unique markers and regulatory changes, thus representing an ‘off-target’ reprogrammed state. The early specification of this state is supported by our gene regulatory network (GRN) inference using CellOracle^37^, suggesting that network reconfiguration is unique to each trajectory and is established early in the reprogramming process. CellTag-multi has the potential to define the molecular features of these early states, offering deeper mechanistic insight into the reprogramming process.

## Foxd2 and Zfp281 drive on- and off-target reprogramming

**Fig. 5:**
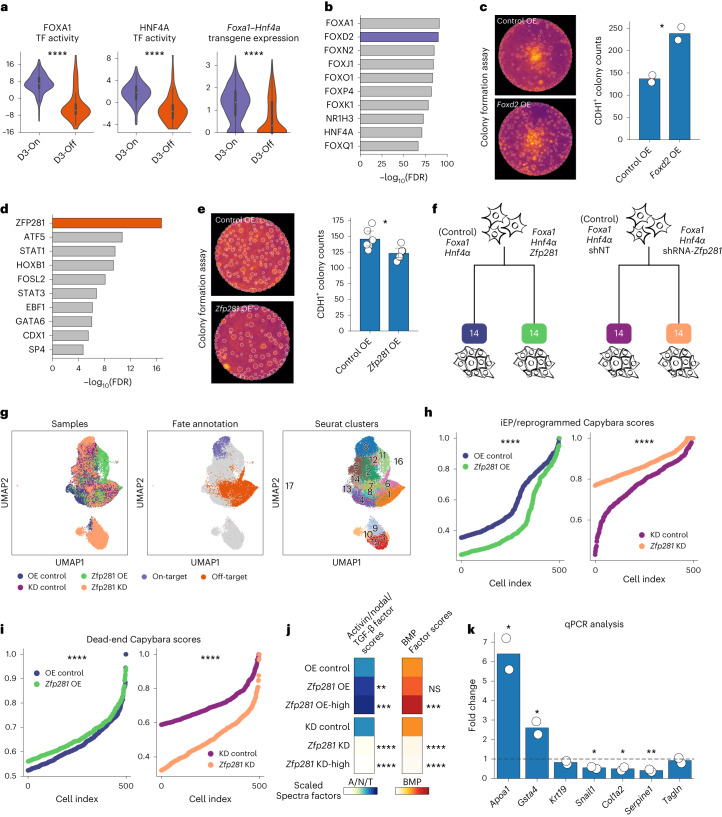
Identification of TF regulators of on-target and off-target reprogramming fate. **a**, Violin plots of FOXA1 and HNF4A TF activities and *Hnf4α–Foxa1* transgene expression across the two fates on day 3 (Mann–Whitney-Wilcoxon test; two-sided; FOXA1 *P* = 1.2 × 10^−^^20^, HNF4A *P* = 4.7 × 10^−^^19^, *Hnf4α–Foxa1* *P* = 1.3 × 10^−^^41^; cell numbers—as indicated in Extended Data Fig. 6d). **b**, Top ten TF activities enriched in on-target destined cells. **c**, Representative images from the *Foxd2* OE colony formation assay (CFA, left); mean CDH1^+^ colony counts in *Foxd2* OE versus standard reprogramming (right, *t*-test, two-sided; *P = 0.025; *n* = 2 biological replicates). **d**, Top ten TF activities enriched in off-target destined cells. **e**, Representative images from the *Zfp281* OE CFA (left); mean CDH1^+^colony counts in the *Zfp281* OE versus standard reprogramming (right; *t*-test, two-sided; *P = 0.017; *n* = 6 biological replicates). **f**, scRNA-seq experiment schematic for *Zfp281* OE and KD during reprogramming. **g**, UMAP for cells from *Zfp281* OE and KD experiments; sample, cell fate and Seurat clusters projected. **h**,**i**, iEP identity scores (**h**) and dead-end identity scores (**i**) across the KD and OE samples compared to controls (Mann–Whitney–Wilcoxon test, two-sided; iEP: OE versus control, *P* = 1.07 × 10^−^^53^; KD versus control, *P* = 2.19 × 10^−^^53^; dead-end: OE versus control, *P* = 1.11 × 10^−^^11^; KD versus control, *P* = 3.26 × 10^−^^120^). **j**, Activin/nodal/TGF and BMP spectra factor scores across control, OE and OE-high cells (top) and control, KD and KD-high cells (bottom). Mean scores are normalized relative to controls. OE-high cells: subset of OE cells with above average *Zfp281* expression. KD-high cells: subset of KD cells with below average *Zfp281* expression (Mann–Whitney–Wilcoxon test, two-sided; ****P < 0.0001; ***P < 0.001; **P < 0.01; NS = *P* > 0.05). **k**, Fold-change in reprogramming and dead-end marker genes expression during TGF-β signaling inhibition compared to control, on day 5 of reprogramming (*t*-test, two-sided; *Apoa1*, *P = 0.02, *Col1a2*, *P = 0.02, *Gsta4*, *P = 0.04, *Serpine1*, *P = 0.009, *Snail1*, *P = 0.01; *n* = 2 technical replicates). Bar plots: error bars: 95% CI. Boxplots—center point, median; box limits, first and third quartiles; whiskers, up to 1.5× interquartile range. CI, confidence interval.

**Extended Data Fig. 9:**
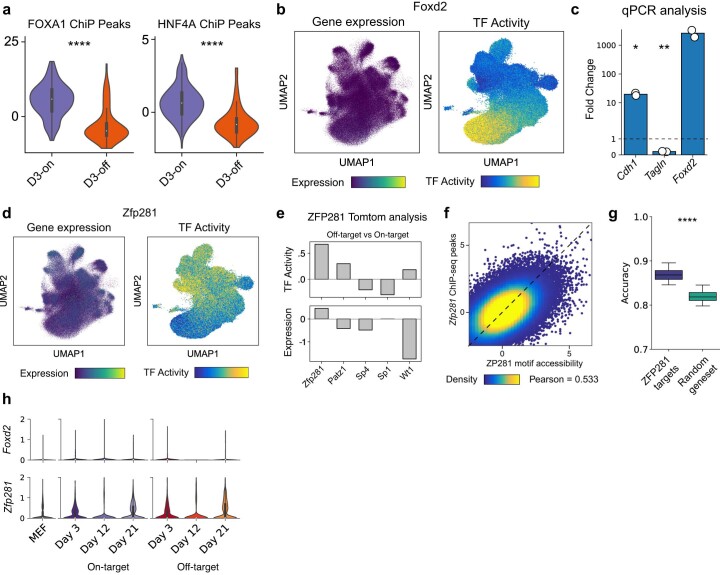
Identification of Zfp281 and Foxd2 as regulators of iEP reprogramming. (**a**) Violin plots comparing accessibility z-scores of FOXA1 and HNF4A genomic binding sites across the two reprogramming fates on Day 3 (Mann Whitney Wilcoxon test, two-sided; p-value: FOXA1 = 1.159e-19, HNF4A = 2.2e-18) suggesting higher on-target binding of the two TFs in the on-target reprogramming lineage on Day 3. (**b**) Projection of *Foxd2* gene expression and FOXD2 TF activity levels on the clone-cell embedding. (**c**) Bar plots showing fold-change in on-target and off-target marker genes (*Cdh1* and *Tagln* respectively) upon *Foxd2* over-expression, compared to a GFP control, on reprogramming day 12 (*t*-test; p-values: *Tagln* = 0.006, *Cdh1* = 0.03; n=2 biological replicates). (**d**) Projection of *Zfp281* gene expression and ZFP281 TF activity levels on the clone-cell embedding. (**e**) Tomtom analysis identified four dead-end enriched TFs with significantly similar motifs to ZFP281. ZFP281 shows the highest enrichment in dead-end cells for both gene expression and TF activity levels across all TF candidates. (**f**) Scatterplot showing correlation between single-cell accessibility of ZFP281 genomic binding sites and ZFP281 motifs (Pearson correlation coefficient = 0.533). (**g**) Boxplot showing significantly higher cell fate prediction accuracy using ZFP281 target genes (1,612 genes) compared to a size matched set of random genes (Mann Whitney Wilcoxon test, two-sided; p-value = 2.248e-09; n=25 accuracy values/boxplot). (**h**) Violin plots showing expression levels of *Foxd2* and *Zfp281* in uninduced MEFs and along the two lineages. All boxplots: center point: median; box limits: first and third quartiles; whiskers: upto 1.5x interquartile range. Panels a and h: Cell numbers – MEF: 10,526; Others – as indicated in Extended Data Fig. 6d.

Higher accessibility of both motifs and genomic targets^53^ of FOXA1 and HNF4A in on-target cells on day 3 suggests significant differences in genomic engagement of the reprogramming TFs between the two fate outcomes (Fig. 5a and Extended Data Fig. 9a). This could, at least in part, be explained by differential expression levels of the *Hnf4α–Foxa1* transgene across the two lineages, with off-target destined cells displaying significantly lower transgene expression (Fig. 5a; Mann–Whitney–Wilcoxon test; *P* = 1.3 × 10^−^^41^). However, we have also previously described an off-target trajectory expressing high transgene levels, suggesting additional mechanisms influencing genomic engagement by the reprogramming TFs^37^.

Outside of FOXA1 and HNF4A, we identified FOXD2 as the top on-target fate-specifying TF candidate (Fig. 5b and Extended Data Fig. 9b). Adding Foxd2 to the Foxa1 and Hnf4α reprogramming cocktail led to increased expression of the iEP marker *Cdh1* and decreased expression of mesenchymal marker *Tagln* on reprogramming day 12 (*t*-test; *Cdh1*, *P* = 0.03; *Tagln*, *P* = 0.006; two biological replicates; two technical replicates; Extended Data Fig. 9c). In addition, colony formation assays showed an increase in the number of CDH1^+^ colonies formed with the addition of Foxd2 to the standard iEP reprogramming cocktail (*t*-test; *P* = 0.045; two biological replicates; Fig. 5c), validating its role in improving on-target fate conversion.

The top off-target-enriched candidate was ZFP281, a zinc finger protein (Fig. 5d and Extended Data Fig. 9d). Zfp281 is a known regulator of cell fate in mouse embryonic stem cells^54^ and promotes epithelial-to-mesenchymal transitions (EMTs)^55^. To further confirm the inferred enrichment of ZFP281 TF activity in off-target fated cells, we performed Tomtom motif similarity analysis^56^ to identify TFs that share a motif similar to ZFP281. We found four other TF motifs that were both significantly similar to the ZFP281 motif (adjusted *P* < 0.05) and were enriched in off-target destined cells. Among these TFs, ZFP281 displayed the highest enrichment in the off-target lineage both in terms of gene expression and TF activity (Extended Data Fig. 9e). Additionally, single-cell accessibility of ZFP281 genomic targets^54^ was positively correlated with inferred ZFP281 TF activity (Pearson’s correlation coefficient = 0.53; Extended Data Fig. 9f) and ZFP281-regulated genes^57^ were significantly more predictive of cell fate as compared to a size-matched set of random genes (Mann–Whitney–Wilcoxon test; *P* = 2.248 × 10^−^^9^; Extended Data Fig. 9g), further confirming its role in off-target fate specification during iEP reprogramming. Notably, both *Zfp281* and *Foxd2* failed to show a strong lineage-specific bias in gene expression levels, highlighting the unique insights offered by multi-omic lineage tracing in the identification of fate-specifying TFs (Extended Data Fig. 9h).

**Extended Data Fig 10:**
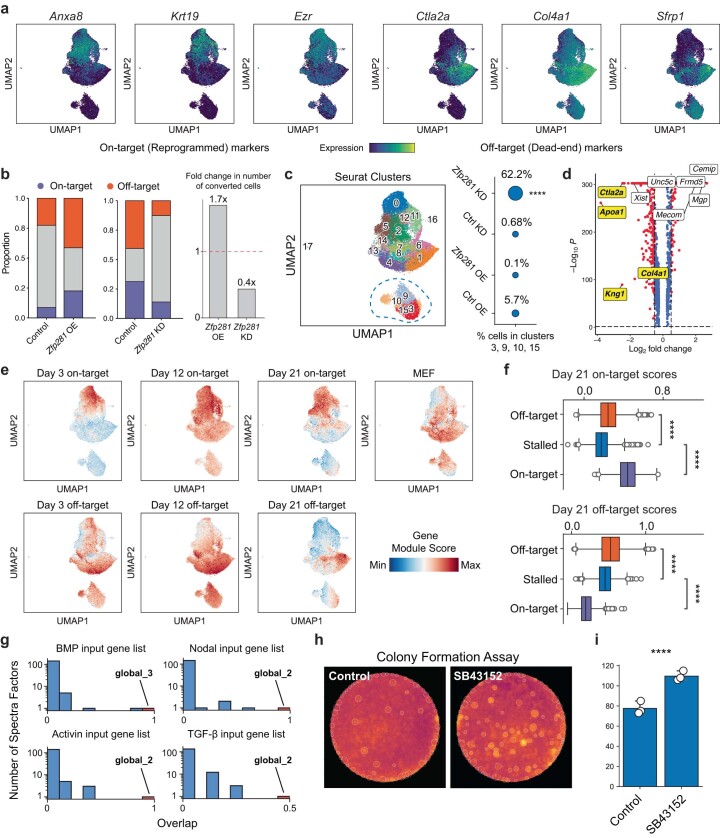
Single-cell analysis of *Zfp281* knockdown and overexpression. (**a**) Projection of key on-target and off-target reprogramming marker genes on the UMAP for *Zfp281* overexpression and knockdown cells. (**b**) (Left Panel) Bar plots showing proportion of on-target and off-target fate cells and (Right Panel) change in total number of reprogrammed cells across the KD and OE experiments. A positive correlation between rate of reprogramming and *Zfp281* expression suggests a role for the TF in promoting fate conversion away from the starting MEF identity. (**c**) (Left Panel) UMAP highlighting a distinct sub-population of cells, likely representing a stalled reprogramming cell state. (Right Panel) Dot plot showing the proportion of each sample in the stalled clusters. Cells from the *Zfp281* KD sample are enriched in the stalled cell states (Permutation test, one-sided; p-value = 0; 100,000 trials). (**d**) Volcano plot showing genes differentially enriched in the stalled cell sub-population (adjusted p-value < 0.05; Benjamini-Hochberg correction, absolute log2 fold-change > 0.5). (**e**) Gene expression module scores for MEF, on-target and off-target marker genes from all three time points, based on the lineage tracing experiment, projected on the UMAP. (**f**) Boxplots comparing module scores for Day 21 off-target, and Day 21 on-target marker genes module scores across stalled cells and the two reprogrammed clusters (Mann Whitney Wilcoxon test, two-sided; **** = p-value < 0.0001; Exact p-values in Supplementary Table 12; Cell numbers – Off-target: 7,069; On-target: 1,706; stalled: 4,726). Boxplots: center line, median; box limits, first and third quartiles; whiskers, 1.5x interquartile range. (**g**) Histograms showing overlap of all learned Spectra factors with each signaling pathway input gene list. BMP input list overlaps maximally with the ‘global_3’ factor (overlap = 1) while Activin, Nodal and TGF-β input lists overlap maximally with the ‘global_2’ factor (overlap = 1 for Activin and Nodal; overlap = 0.5 for TGF-β). (**h**) Representative images from the SB43152 colony formation assay; (**i**) Mean CDH1-positive colony counts in cells cultured in presence of SB43152 compared to a standard reprogramming experiment (*t*-test, two-sided; p-value = 2.26e-3; n = 3 biological replicates). Error bars represent 95% CI.

Indeed, inclusion of Zfp281 along with Foxa1 and Hnf4α in the reprogramming cocktail resulted in a moderate but statistically significant reduction in the number of CDH1^+^ colonies (*t*-test; *P* = 0.017; Fig. 5e). To further characterize the role of Zfp281 in reprogramming, we performed both OE- and shRNA-mediated knockdown (KD) of Zfp281, along with respective control samples, and profiled cells with scRNA-seq on reprogramming day 14 (Fig. 5f,g and Extended Data Fig. 10a). We found that the rate of reprogramming (both on-target and off-target) increased with increasing *Zfp281* expression (Extended Data Fig. 10b), suggesting a role for Zfp281 in accelerating fate conversion in iEP reprogramming. Moreover, we identified a distinct subpopulation of cells, predominantly consisting of Zfp281 KD cells that were depleted for expression of key markers of both on-target and off-target reprogramming such as *Apoa1* and *Ctla2a* (Extended Data Fig. 10c,d and Supplementary Table 9). These cells were enriched for genes associated with negative regulation of mesenchymal cell migration (Supplementary Table 9), reinforcing Zfp281’s putative role in mesenchymal fate specification. Additionally, they were depleted for expression of both off-target and on-target markers genes from day 21 (obtained from our lineage analysis; Extended Data Fig. 10e,f) and thus likely represent a ‘stalled’ cell state due to reduced *Zfp281* expression levels. Despite its acceleration of cell fate conversion broadly, we found that Zfp281 shifted the identity of reprogrammed cells away from an iEP-like state and toward a dead-end/off-target-like state consistently across the OE and KD experiments (Fig. 5h,i), confirming a role for Zfp281 in biasing cells toward an off-target fate, as suggested by our lineage-tracing analysis. This finding also explains the reduced number of CDH1^+^ colonies observed in our colony formation assay, despite the increase in the total number of on-target reprogrammed cells upon Zfp281 OE.

TGF-β and nodal are two closely related signaling pathways, both of which are downstream effectors of Zfp281 (refs. ^50,58^). TGF-β has previously been reported to have a key role in EMT^59^. Because upregulation of mesenchymal genes and failure to epithelialize are key hallmarks of the off-target reprogramming fate, we assessed potential changes in these signaling pathways upon OE/KD of *Zfp281*. We used Spectra^60^ to compute single-cell pathway scores for the following four closely related signaling pathways: TGF-β, nodal, activin and bone morphogenic protein (BMP; Methods). Spectra is a supervised factor analysis method that uses user-defined global and cluster-specific gene sets to produce gene programs (factors) for a given dataset. We applied Spectra to our Zfp281 OE and KD datasets, providing lists of ligand–receptor pairs for each of the four pathways^61^ as global input gene sets and marker genes from our lineage analysis as cluster-specific input gene sets (Methods). Comparing Spectra factors to our input gene lists, we identified a ‘BMP factor’ and an ‘activin/nodal/TGF factor’ common to the activin, nodal and TGF-β signaling pathways (Extended Data Fig. 10g and Supplementary Table 10; Methods). The activin/nodal/TGF factor scores increased significantly in *Zfp281* OE cells and decreased in *Zfp281* KD cells, relative to respective controls (Fig. 5j), suggesting active regulation of at least one of the three pathways by Zfp281. Similar changes were observed in BMP factor scores upon Zfp281 OE/KD (Fig. 5j). Indeed, inclusion of SB431542 (ref. ^62^)—a small molecule inhibitor of TGF-β, activin and nodal signaling pathways—led to an increase in expression of reprogramming marker genes *Apoa1* and *Gsta4* and a decrease in expression of mesenchymal/off-target genes such as *Serpine1, Snail1* and *Col1a2* (Fig. 5k). This was accompanied by a significant increase in the number of CDH1^+^ colonies during reprogramming (Extended Data Fig. 10h,i) suggesting a crucial role for these pathways in determining fate outcome during iEP reprogramming.

## Discussion

Here we have presented CellTag-multi, a method for independent scLT across scRNA-seq and scATAC-seq assays. In the context of hematopoiesis, we have used CellTag-multi to map transcriptional and epigenomic states of progenitor cells and link them to clonal fate, recapitulating enrichment of known lineage-specific cell-state signatures across progenitor populations. With chromatin state, we showed that lineage-specific epigenetic priming is associated with changes in accessibility of known fate-specifying TF motifs and that such changes occur primarily in the regions of the genome distal to promoters. Previous analysis has demonstrated the inability of early transcriptional state alone in predicting cell fate and posited a role for alternate cell-state modalities^12^. By exploiting multi-omic clonal relationships, we demonstrated that the predictability of cell fate from state is significantly improved when both early transcriptional and epigenomic state are considered, as opposed to either modality individually, suggesting that the RNA and ATAC modalities consist of nonredundant and highly complementary state information.

Our application of CellTag-multi to the less characterized paradigm of iEP reprogramming generated similar observations, where multi-omic clonal data captured in the early stages of fate conversion are highly predictive of reprogramming outcome. Again, fate-specifying epigenetic changes during early stages of differentiation are dominated by changes in distal regulatory regions of the epigenome. Furthermore, we have been able to molecularly characterize the ‘dead-end’ state as a highly proliferative, mesenchymal-like cell state, representing an ‘off-target’ reprogrammed fate. Indeed, a similar state has been reported in direct reprogramming of mesenchymal stromal cells to induced hepatocytes, revealing the appearance of *Acta2*-expressing mesenchymal cells during the reprogramming process^63^. Outside of the hepatic lineage, off-target identities have been reported in other reprogramming paradigms^34,64^, suggesting that this may be a more general feature of lineage reprogramming.

Our multi-omic lineage tracing demonstrates the establishment of on-target and off-target trajectories from early stages, supported by our earlier transcriptome-based lineage tracing of iEP reprogramming^7^ and GRN inference^37^. However, given the single-modality capture of relatively few clones in that earlier study, we were not able to comprehensively characterize early molecular states. Here the collection of ground truth data on lineage, transcriptome and epigenome has allowed us to better characterize these distinctive early states, enabling mechanistic insights into reprogramming. We have shown crucial early differences in gene regulation that lead to distinct reprogramming outcomes. Specifically, we have identified and experimentally validated that Foxd2 promotes successful reprogramming, while Zfp281 activity leads to engagement with an off-target trajectory. Differences in reprogramming TF levels may account for these early differences. However, lower levels of exogenous TF expression do not simply lead to reprogramming failure, as the off-target fate is molecularly unique from fibroblasts and could be considered a reprogramming byproduct in itself. These results suggest that the stoichiometry of TF OE in these reprogramming models may offer further insight into how TFs control cell identity. Single-cell analysis of TF binding could provide further insights into the role of differential binding of the two reprogramming TFs in specifying off-target fate.

Our recovery of Foxd2 and Zfp281 as regulators of early-stage reprogramming was not possible from differential gene expression analysis alone, demonstrating the utility of CellTag-multi. Moreover, off-target enrichment of Zfp281’s TF activity from early stages of reprogramming despite any lineage-specific bias in its expression levels could indicate a role for secondary mechanisms such as cofactor binding or post-translational modifications in modulating the TF’s function. Although not a direct perturbation of genome-wide accessibility of ZFP281 binding sites, our OE and KD experiments validate this observation. From our experimental validation, we found that KD of Zfp281 expands a population of cells in a ‘stalled’ state, where they fail to extinguish fibroblast gene expression while upregulating off-target cells. Conversely, OE of Zfp281 helps accelerate fate conversion, resulting in a considerable increase in reprogramming efficiency. However, Zfp281 still draws the reprogrammed cells toward an off-target, mesenchymal-like state. A role for this TF in driving broad mesenchymal expression programs, including components of the TGF-β and nodal signaling pathways, has recently been described^50,58^. Here we demonstrate that the inhibition of related signaling pathways—TGF-β, activin and nodal—enhances on-target marker expression while decreasing off-target gene expression. These results suggest a potential strategy to enhance on-target reprogramming, where Zfp281 expression can help erase the starting cell identity while blocking downstream TGF-β signaling might prohibit entry onto the off-target trajectory.

Altogether, the data we present here across two distinct biological systems demonstrate that lineage-specific capture of gene expression and chromatin accessibility provides rich information on gene regulation, offering unique mechanistic insights into the specification and maintenance of cell identity. More widely, scLT has revealed distinct, clonally heritable transcriptional states across various biological systems^65–67^. These phenotypic differences, arising from seemingly nongenetic sources, have strong biological implications. For example, clonal variability in cell state has been shown to impact malignant clonal expansion and efficacy of drug treatment in cancer cells^65,67^. Elsewhere, clustered regularly interspaced short palindromic repeats-based systems have been used to create mutable barcodes to allow multilevel lineage recording without the need for successive rounds of cell labeling^68,69^. Given its versatility and ease of use, we envision that CellTag-multi can be readily applied to such biological questions and use cases.

Finally, we have developed CellTag-multi to work independently with scRNA-seq and scATAC-seq, as existing single-cell methods that co-assay multiple modalities from the same cell^70–73^ can suffer from lower data quality compared to methods that profile each modality individually. Furthermore, enabling the capture of lineage in parallel with chromatin accessibility provides users with additional flexibility for experimental design. Advances in single-cell technologies are allowing the measurement of an ever-increasing number of cellular modalities such as DNA methylation and histone state. A similar expansion in multi-omic lineage-tracing assays will enable deeper mechanistic insight into the regulation of cell identity and clonal heritability of cell state. CellTag-multi, with its cell lineage readout alongside gene expression and chromatin accessibility, paves the way for the development of such multi-omic, scLT methods.

## Methods

### Isolation of mouse LSK cells

LSK cells were obtained using a previously described protocol^12^. Adult mice were euthanized, and bone marrow was extracted and passed through a 70 µm filter. Cells were centrifuged at 300*g* for 10 min at 4 °C, resuspended in EasySep buffer (STEMCELL, 20144) at 100 million cells per ml and differentiated cells were removed using the EasySep lineage depletion kit (STEMCELL, 19856). Cells were stained for Sca1 (Sca1-AF488; BioLegend clone D7) and cKit (CD117-PE; BioLegend clone 2B8) and sorted using the MoFlo Cell Sorter (Beckman Coulter) with a 130 µm nozzle.

### Mice and derivation of MEFs

MEFs were derived from embryonic day (E)13.5 C57BL/6J embryos (Jackson Laboratory, 000664). Heads and visceral organs were removed, and the remaining tissue was minced with a razor blade, dissociated in a mixture of 0.05% trypsin and 0.25% collagenase IV (Life Technologies) at 37 °C for 15 min and the cell slurry was passed through a 70-μM filter to remove debris. Cells were washed and plated on 0.1% gelatin-coated plates, in DMEM supplemented with 10% FBS (Gibco), 2 mM l-glutamine and 50 mM β-mercaptoethanol (Life Technologies). All animal procedures were based on animal care guidelines approved by the Institutional Animal Care and Use Committee at Washington University in St. Louis.

### Lentivirus and retrovirus production

Lentiviral particles were produced by transfecting 293T-17 cells (American Type Culture Collection: CRL-11268) with the pSMAL-CellTag construct (see Supplementary Experimental Methods, CellTag-multi library synthesis), along with packaging constructs pCMV-dR8.2 dvpr (Addgene, 8455), and pCMV-VSVG (Addgene, 8454). Retroviral particles for the bicistronic Hnf4a-T2A-Foxa1 construct were produced as previously described^7^. Virus was collected 48 h and 72 h after transfection and applied to cells immediately following filtering through a low-protein binding 0.45-μm filter. Wherever applicable, the virus was concentrated using ultracentrifugation. In total, 20 ml of filtered viral supernatant was centrifuged at 50,000*g* for 2.5 h at 4 °C, the supernatant was removed and the virus was resuspended in 100 µl of DMEM and stored at −80 °C.

### Section 1

#### Species-mixing experiment

For the species-mixing experiment, mouse iEP-LT cells were tagged with CellTag-multi library, containing the barcode pattern (N)_3_GT(N)_3_CT(N)_3_AG(N)_3_TG(N)_3_CA(N)_3_ and human HEK 293T cells with CellTag-multi-v0 library, containing the barcode pattern (N)_5_GTA(N)_5_CCT(N)_5_ATC(N)_5_GAT(N)_5_. Nuclei were isolated from both using the 10X Genomics scATAC-seq nuclei isolation protocol (CG000169) and mixed in a 1:1 ratio. The sample was processed using the standard 10X Genomics scATAC-seq library preparation (v1 kit) with modifications to capture CellTags (Supplementary Methods). Single-cell libraries were sequenced on an Illumina NextSeq-500, and sequencing data were aligned to a mixed species reference using CellRanger. The aligned BAM file was used for downstream analysis.

Reads matching v0 or v1 CellTags were parsed from the mixed species single-cell aligned BAM file. Each cell barcode was assigned to one of four categories, based on CellRanger-ATAC species assignments—human, mouse, doublet, noncell; the distribution of v0 and v1 reads was assessed across the four categories. Cells with fewer than two CellTag reads across both libraries were discarded, and the remaining cells were plotted on a species-mixing plot. We quantified interspecies cross-talk of CellTags, by calculating the percent of cells, with at least two CellTag reads per cell, having less than 95% of CellTag reads originating from the correct, species-specific CellTag library.

#### Assessing the effect of isRT on chromatin accessibility signal

We compared the effect of introducing an isRT step on scATAC-seq data quality. For this, two single-cell ATAC libraries were prepared with CellTagged HEK 293T cells using either the original 10X Genomics scATAC library preparation protocol (Original) or our modified method (Modified). Sequencing data from both were processed with ArchR^74^, dimensionally reduced using latent semantic indexing, clustered using Louvain clustering, and peaks were identified across samples. Normalized peak counts (counts per million) were calculated for each sample and plotted on a scatterplot, and the Pearson correlation coefficient was calculated to quantify the similarity between the genome-wide accessibility signal of the two samples.

#### Analysis of clones in expanded reprogramming fibroblasts

A subset of the data obtained from our reprogramming dataset (described in Section 3) from days 12 and 21 was used for this analysis. Clones were identified following the workflow described in Supplementary Methods. CellTag abundance was calculated for each CellTag as the percent of cells containing that CellTag after filtering and binarization. Browser tracks depicting single-cell accessibility fragments were plotted using ArchR. Gene expression and gene score values were averaged on a clonal level. Spearman correlation coefficients were calculated between clonal gene expression and gene score both within (intraclonal) and across clones (interclonal).

#### Comparison of scRNA-seq, scATAC-seq and 10X multiome-based CellTag capture

The 10X Genomics RNA + ATAC Multiome libraries were prepared from reprogrammed cells from day 21 of replicate two of our reprogramming datasets (Section 3) and compared to a similar number of day 21 cells profiled with scRNA-seq and scATAC-seq for the same replicate. For multiome samples, CellTag amplicon libraries were obtained using cDNA generated during the scRNA part of the library prep (Supplementary Methods, CellTag-RNA PCR) but with 15 cycles of sample index PCR as opposed to the standard 11 and sequenced on a NextSeq-500. Multiome CellTag reads were processed exactly like scRNA-seq CellTag reads. CellTag library complexity was calculated as the total number of unique Cell Barcode—UMI—CellTag barcode combinations detected in each CellTag amplicon library. This analysis was omitted for scATAC-seq CellTag reads due to lack of UMIs. To compare sequencing quality metrics (fraction of reads in peaks and percent mitochondrial reads) multiome, scATAC-seq and scRNA-seq were downsampled to an equal sequencing depth per cell.

### Section 2

#### Lineage tracing during in vitro mouse hematopoiesis

LSK cells were purified as described above, counted and 5,500 cells were added to a 96-well U-bottom suspension culture plate (GenClone, 25-224) and allowed to recover in broad myeloid differentiation media^12^ consisting of serum-free expansion medium (STEMCELL), penicillin–streptomycin (Pen–Strep), interleukin (IL)-3 (PeproTech, 213-13; 20 ng ml^−1^), FLT3-L (PeproTech, 250-31L; 50 ng ml^−1^), IL-11 (PeproTech, 220-11; 50 ng ml^−1^), IL-5 (PeproTech, 215-15; 10 ng ml^−1^), erythropoietin (PeproTech, 100-64; 3 U ml^−1^), thrombopoietin (PeproTech, 315-14; 50 ng ml^−1^) and mouse stem cell factor (R&D Systems, Q78ED8; 50 ng ml^−1^) and IL-6 (R&D Systems, 406-ML-005; 10 ng ml^−1^) at 37 °C for 2 h.

To allow clone tracking, cells were transduced for 2 d with 10 µl of concentrated CellTag-multi virus (~25 k unique CellTag sequences) in 100 µl differentiation media, in the presence of 6 µg ml^−1^ diethylaminoethyl–Dextran after spin-fection at 800*g* for 90 min at 37 °C. Sixty hours (2.5 d) after the start of the experiment, 50% of the cells were collected for single-cell profiling. The remaining cells were split into two technical replicates and replated in fresh differentiation media. Finally, all the cells were collected on day 5 for single-cell profiling. At each time point, cells for single-cell profiling were split equally between scRNA-seq (single-index v3 kit) and scATAC-seq (v1 kit) with modifications to capture CellTags (Supplementary Methods). RNA libraries were sequenced on an Illumina NovaSeq-6000 and computationally dehopped. RNA CellTag amplicons were sequenced on an Illumina NextSeq-500. CellTag and transcriptome read files for each sample were processed together using CellRanger, using a custom mm10 reference containing GFP, to produce one BAM file per sample. ATAC libraries containing both accessible chromatin and CellTag fragments were sequenced on an Illumina NextSeq-500 and processed using CellRanger-ATAC, using the default mm10 reference genome. Aligned BAM files from both modalities were used for CellTag processing^75^, and other CellRanger and CellRanger-ATAC outputs were used for downstream single-cell analyses.

#### Basic single-cell and clonal analysis of the hematopoiesis dataset

scRNA-seq count matrices were processed using Seurat. Low-quality cells with high mitochondrial reads, low UMIs and features per cell were removed, and the two time points were integrated using SCTransform, dimensionally reduced using principle component analysis (PCA) and clustered using Louvain clustering. Fragments files from scATAC-seq samples were processed using ArchR v1.0.1. Valid cell barcodes (from CellRanger-ATAC) passing default ArchR quality filters were retained. Cells were dimensionally reduced using iterative LSI and clustered using Louvain clustering. Cell types were annotated using known hematopoietic marker genes in scRNA-seq^12^. Cell-type labels were transferred to scATAC-seq cells using Seurat label transfer, and annotations were manually verified by inspecting the accessibility of marker genes (gene activity scores). For RNA–ATAC co-embedding, scRNA-seq gene expression matrix and imputed^76^ scATAC-seq gene score matrix were used as input to the RunCCA function in Seurat. A union set of the top 5,000 HVG from each dataset was used for this co-embedding.

For clone calling, the cell × CellTag UMI (for RNA) and read (for ATAC) count matrices were obtained. The RNA matrix was binarized at a threshold of >1 UMI count per cell, and cells with 2–25 CellTags were retained. The ATAC matrix was binarized at a threshold of >1 read count per cell, and cells with 1–25 CellTags were retained. The two filtered matrices were merged, and the cell–cell Jaccard similarity matrix was computed and thresholded at 0.6 (for cell pairs within the same modality) and 0.5 (for cell pairs across modalities). The final thresholded matrix was used to identify clones across the entire dataset. Clone-cell embedding was computed as described in Supplementary Methods, and ForceAtlas2 was used to jointly visualize clones and cells. For single-modality clonal analysis, cell × CellTag matrices for each modality were processed separately with the same thresholds as above. A Jaccard threshold of 0.5 was used for ATAC clone calling and 0.6 was used for RNA clone calling.

For homoplasy simulation, we used a population size of 5,500 cells, 1–25 CellTags/cell and an average MOI of 3.4. A total of 100 simulations were performed, and average values were reported.

#### Inference of lineage hierarchies using scRNA and scATAC lineage data

Lineage hierarchies were obtained using CoSpar^77^ using the cospar.pp.initialize_adata_object function PCA for RNA and LSI from ATAC data as input embeddings. The corresponding clone tables were added to each object using the cospar.pp.get_X_clone function. Finally, RNA and ATAC transition maps were computed using the cospar.tmap.infer_Tmap_from_multitime_clones function and fate hierarchies were obtained using cospar.tl.fate_hierarchy for major hematopoietic fates, as indicated in Fig. 2c,d. Finally, CoSpar inferred trees were converted to Cassiopeia^78^ objects, and the RF distance metric was calculated using the cassiopeia.critique.robinson_foulds function.

To assess changes in inferred fate hierarchies at different dataset sizes, the RNA object was subsampled to either 10,000, 20,000 or 40,000 cells. For each subset, fate hierarchies were inferred independently as described above and RF distance between the full dataset tree and subsampled dataset trees were calculated.

#### State–fate linkage in hematopoiesis

To link cell state with fate, each clone was assigned a fate label based on the predominant fate among its day 5 siblings. Scarce lineages were grouped for similarity (Ery/Meg, Baso/Eos/Mast, DCs). Clones labeled as transitions or progenitors were excluded from the state–fate analysis, unless specified. Fate bias scores were determined as the percentage of day 5 siblings belonging to the annotated fate label.

Each clone was divided into up to four subclones based on the time point and assay of each sibling, and the clone-cell embedding was recalculated. The overlap between RNA and ATAC subclones across the two single-cell modalities was assessed within each ‘fate potential’ group using the Wasserstein distance metric with a 30-dimensional UMAP-based embedding of the subclone nodes.

To evaluate if state subclones closer to the periphery of a ‘fate potential’ group exhibited less fate bias, we introduced a closeness metric. This metric measures the minimum distance of a state subclone from the centroid of an alternative fate potential group. A higher closeness metric indicates that a state subclone is further away from the centroids of other fate potential groups. We then plotted the relationship between the closeness metric and fate bias using a percentile plot, with the *x* axis representing the percentile rank for the closeness metric and the *y* axis showing the mean fate bias scores for state subclones passing that percentile rank.

To characterize functional priming of cell state, day 2.5 state siblings in each fate potential group were compared to the rest in gene expression and TF activity space. For scRNA-seq features, we used residuals obtained for the top 3,000 HVG after SCTransform normalization in Seurat. For scATAC-seq features, we used chromVAR-derived TF activity *z* scores (default mouse motif set in ArchR—884 TF motifs). Correction for multiple hypothesis testing was performed using the Benjamini–Hochberg method, setting the FDR threshold for significance at 0.05, unless otherwise specified. Additionally, ‘biological process’ GO term enrichment analysis was performed for the top 100 gene markers for each fate potential group using the PANTHER classification system^79^ (release 17.0; http://geneontology.org/), and terms with FDR < 0.01 were reported in Supplementary Table 4.

#### Fate prediction from cell state using machine learning

We performed state–fate machine learning to predict cell fate from the early state. A machine-learning classifier used single-cell features X of day 2.5 cells to predict discrete clonal fate labels (for example, ‘progenitor’, ‘monocyte’ and ‘neutrophil’). For RNA only, we used residuals of the top 3,000 genes. For ATAC only, we used TF activity *z* scores (k-nn imputation with *k* = 20). For RNA + ATAC, we paired siblings and used combined features. Repeated Stratified *k*-fold cross-validation (n_splits = 5, n_repeats = 5) was used for analysis, resulting in 25 accuracy/weighted F1 score values. Results are depicted using boxplots.

For each machine-learning task, we tested a panel of classifier architectures, logistic regression, LightGBM and random forest. Each was trained and evaluated using the procedure described above. Hyperparameter tuning was performed for each and the following values were tested:

- Random Forest: n_estimators: [100, 300, 1000], max_depth: [10, 50, None], min_samples_leaf: [1,2,4], bootstrap: [True, False]
- LightGBM: num_leaves: [7,15,31,80], max_depth: [5,9,30], min_data_in_leaf: [20,40,80], bagging_fraction: [0.8,1], bagging_freq: [3], feature_fraction: [0.1, 0.9]
- Logistic Regression: penalty: [‘l2’, ‘none’], C: np.logspace(-4, 4, 20), solver: [‘lbfgs’,‘newton-cg’,‘saga’], max_iter: [1000]

The Python library ‘scikit-learn’ was used for all machine-learning analysis.

#### Fate prediction using TF activities derived from distal, intronic, exonic and promoter peak sets

ATAC peaks were categorized (intronic, exonic, promoter or distal) using default ArchR definitions. TF activity scores were calculated for each peak set independently and used for the state–fate prediction as described before. To test if performance variation was due to different peak numbers, all sets were randomly subsampled to 8,823 peaks (exonic set size), and state–fate prediction was done using these new scores.

#### SHAP analysis

The ‘SHAP’ python package was used for SHAP analysis to interpret trained machine-learning models. SHAP values were calculated using the TreeExplainer function from the package for trained random forest models. For each input feature and fate label, SHAP values were computed using each data point in the 25 test sets (n_splits × n_repeats), resulting in 5 SHAP values per data point per feature to average out any outliers caused by model training artifacts.

Feature importance scores were then determined for each input feature regarding the prediction of each fate label by calculating the mean of absolute SHAP values for each feature-fate combination. To identify features positively or negatively correlated with the prediction of a fate label, SHAP correlation was performed. For each input feature, the Pearson correlation coefficient between its values (expression/TF activity) and its SHAP values for a specific fate was computed, resulting in one correlation value per feature per fate.

### Section 3

#### Lineage tracing during iEP reprogramming

Cryo-preserved P0 MEFs were thawed and seeded on 0.1% gelatin-coated six-well plates, in DMEM supplemented with 10% FBS, 2 mM l-glutamine and 50 mM β-mercaptoethanol (Life Technologies) and Pen–Strep at a density of 30,000 cells per well. After overnight recovery at 37 °C, cells were transduced every 12 h for 2 d, with fresh Hnf4α-T2A-Foxa1 retrovirus in the presence of 4 μg ml^−1^ protamine sulfate (Sigma-Aldrich). During the last round of transduction, the retroviral mixture was supplemented with CellTag-multi lentiviral library to initiate clone tracking. On day 0 of reprogramming, cell culture media was changed to hepato-medium (DMEM:F-12, supplemented with 10% FBS, 1 μg ml^−1^ insulin (Sigma-Aldrich), 100 nM dexamethasone (Sigma-Aldrich), 10 mM nicotinamide (Sigma-Aldrich), 2 mM l-glutamine, 50 mM β-mercaptoethanol (Life Technologies) and Pen–Strep, containing 20 ng ml^−1^ epidermal growth factor (Sigma-Aldrich)). After 72 h (day 3 of reprogramming), cells were dissociated, two-thirds of the cells were collected for single-cell sequencing and the remaining cells were replated on six-well plates coated with 5 μg cm^−^^2^ type I rat collagen (Gibco, A1048301). Two additional samples were collected on days 11 and 21 for single-cell sequencing. We used the 10X Genomics 3′ scRNA kit (v3.1; dual index) and the scATAC kit (v1.1) for single-cell profiling. This experiment was performed in two biological replicates.

CellTag PCR was performed for all scRNA-seq and scATAC-seq libraries, as described in Supplementary Methods. scRNA-seq and scATAC-seq libraries were sequenced on an Illumina NovaSeq-6000. CellTag amplicon libraries were sequenced on an Illumina NextSeq-500 to avoid any index hopping-related artifacts.

#### Basic single-cell and clonal analysis of the direct reprogramming dataset

scRNA-seq count matrices were processed using Seurat. Low-quality cells with high mitochondrial reads, low UMIs and features per cell were removed, and all time points and biological replicates were integrated, dimensionally reduced using PCA and clustered using Louvain clustering. Single-cell identity scores were obtained using Capybara, using Fibroblasts (MEFs), and reprogrammed cells and dead-end trajectory references from a previous dataset^7^. Cells from days 12 and 21 were subsetted, reclustered and annotated as ‘reprogrammed’, ‘dead-end’ or ‘transition’ based on these cell identity scores and marker gene expression. scATAC cells were processed exactly as the LSK dataset. Cells were annotated as ‘reprogrammed’, ‘dead-end’ or ‘transition’ based on marker gene accessibility. For RNA–ATAC co-embedding, scRNA-seq gene expression matrix and imputed^76^ scATAC-seq gene score matrix were used as input to the RunCCA function in Seurat. A union set of the top 2,000 HVG from each dataset was used for this co-embedding.

For clone calling, cell × CellTag UMI (for RNA) and read (for ATAC) count matrices were obtained for each modality. The RNA matrix was binarized at a threshold of more than one UMI count, and cells with 1–25 CellTags were retained. The ATAC matrix was binarized at a threshold of more than one read count, and cells with 1–25 CellTags were retained. To reduce false-positive rates, highly abundant single-CellTag signatures (single-CellTag signatures that were also present in multi-CellTag signatures) were removed from our analysis. The two filtered matrices were merged, and cell–cell Jaccard similarity matrix was computed and thresholded at 0.6. The final thresholded matrix was used to identify clones across the entire dataset. Clone-cell embedding was computed (Supplementary Methods), and the UMAP algorithm was used to jointly visualize clones and cells.

For homoplasy simulation, we used a population size of 30,000 cells, 1–25 CellTags/cell and an average MOI of 2.25. Consistent with our clonal analysis, simulated single-CellTag signatures that were also present in simulated multi-CellTag signatures were excluded from homoplasy analysis. A total of 100 simulations were performed, and average values were reported. True/observed rate of homoplasy was calculated by comparing CellTag signatures of single cells across the two biological replicates.

#### State–fate analysis for the direct reprogramming dataset

Clones were annotated with one of the following three fates: reprogrammed, transition or dead-end, based on the modal cell type among fate siblings. Clonal fate bias scores were calculated as the percentage of fate siblings (days 12 and 21) belonging to the annotated fate label. Alluvial plots were constructed using the ggAlluvial R package. State–fate machine-learning analysis was performed as in the ‘Fate prediction from cell state using machine learning’ section to predict ‘reprogrammed’ or ‘dead-end’ fates.

CellRank analysis was performed on a 40,000-cell subset of the scRNA-seq dataset due to scalability limitations. For feature enrichment analysis, day 3 siblings in state–fate clones were grouped by fate. Both on-target and off-target cell groups were expanded using k-nearest neighbors (*k* = 5) for peak and TF activity comparisons. TF activity results were further refined by discarding TFs with low gene score-TF activity correlation (<0.3). Motif enrichment analysis was performed using the HOMER package^80^ on on-target and off-target DERs with MEF DERs as background. Genomic regions annotated as dELS, pELS, dELS, CTCF-bound or pELS, CTCF-bound in the SCREEN database^42^ were used for enrichment analysis.

The FigR^41^ package was used for peak-to-gene linkage analysis. Optimal matching was used to pair RNA and ATAC cells from the same time points, followed by the runGenePeakcorr function to identify peak–gene pairs. Peak–gene pairs with an adjusted *P* value < 0.05 were retained. FOXA1 and HNF4A chromatin immunoprecipitation followed by sequencing (ChIP–seq) peaks from day 2 of reprogramming were obtained^53^ and added as custom annotations in ArchR. Single-cell accessibility *z* scores for each peak set were computed using the addDeviationsMatrix function in ArchR.

#### Computational analysis related to ZFP281 motifs

Tomtom analysis^56^ from the MEME-ChIP package was used to find highly similar motifs to *Zfp281*. The *Zfp281* position frequency matrix was obtained from ArchR and used as input to the Tomtom web interface. Highly correlated TF motifs with *q* value less than 0.05 were obtained, and these were further subsetted for TF activities enriched in off-target destined cells resulting in a total of four TF motifs for comparison with *Zfp281*. ZFP281 ChIP–seq peaks were obtained^54^, and single-cell accessibility *z* scores were computed using the addDeviationsMatrix function in ArchR. ZFP281 gene targets^57^ were used as inputs for a state–fate prediction model, which was trained and evaluated as described above and compared to a sized-matched set of random genes.

#### Plasmid cloning related to Foxd2 and Zfp281 experiments

Nontargeting shRNA construct was obtained from Sigma-Aldrich (SHC202; pLKO.5-puro Control Plasmid). Zfp281 targeting shRNA gene was obtained from Sigma-Aldrich (clone ID: TRCN0000255746) and cloned into the pLKO.5-puro lentiviral construct (Sigma-Aldrich, SHC201). For OE, cDNA fragments were cloned in the pGCDNsam retroviral construct. *Zfp281* cDNA was obtained from OriGene (MC205914) and *Foxd2* cDNA was RT from RNA obtained from long-term iEP cells.

#### Reprogramming with Foxd2 and Zfp281 perturbations

Reprogramming was performed as described above, with the following modifications. For OE, cells were transduced with a 1:1 mixture of *Foxd2/Zfp281* retrovirus and *Hnf4α–Foxa1* reprogramming retrovirus every 12 h for 2 d. Control cells were transduced with a 1:1 mixture of a GFP control retrovirus and *Hnf4α–Foxa1* reprogramming retrovirus for the same amount of time. For KD, cells were transduced with the nontargeting control/*Zfp281*–shRNA lentivirus every 12 h for 1 d after the 2-d *Hnf4α–Foxa1* retroviral transduction was completed.

#### Single-cell analysis for Foxd2 and Zfp281 experiments

scRNA-seq libraries were prepared for all four samples (*Zfp281* OE, OE control, *Zfp281* KD and KD control) and sequenced on a Nextseq-500. Count matrices were generated and integrated using CellRanger count and aggr commands and processed using Seurat. Quality filtering was performed to remove cells with high mitochondrial reads and low UMIs and genes per cell. Cells were dimensionally reduced using PCA, cell cycle regressed, clustered using Louvain clustering and visualized using UMAP. Capybara identity scores were calculated as described in the ‘Basic single-cell and clonal analysis of the direct reprogramming dataset’ section above. Markers for each lineage across time points and uninduced MEFs were obtained (log_2_(fold change) > 0.7, adjusted *P* < 0.05) and used for gene module scoring for all four samples. Cell clusters enriched with on-target or off-target markers were annotated with the respective fates, and GO analysis was performed as described above (‘State–fate linkage in hematopoiesis’).

#### Spectra analysis for signaling pathways

Mouse-specific ligand–receptor pairs for each pathway were downloaded from the CellChat database. Top 25 genes positively associated with TGF-β signaling from the pROGENY^81^ database were also obtained. These gene lists were provided as global gene sets in Spectra. For cluster-specific factor fitting, seven gene lists enriched along the on-target and off-target reprogramming lineages at each time point and uninduced MEFs were used. Spectra model fitting was done with *λ* = 0.01, and resulting factor lists were compared to input gene lists to identify a BMP signaling factor and an activin/nodal/TGF-signaling factor.

#### Colony formation assays

Colony formation assays were performed as previously described^7^. Reprogramming cells were seeded at low plating density in collagen-coated six-well plates within the first 4 d and allowed to form colonies over 2 weeks of reprogramming. Following this, cells were fixed using 4% paraformaldehyde, permeabilized using 0.1% Triton-X and processed for CDH1 (E-cadherin) staining using the VIP peroxidase substrate kit (Vector Laboratories, SK4600) and anti-mouse E-cadherin primary antibody (BD Biosciences; 1:100). Stained colonies were imaged using a flatbed scanner and quantified using the following script: https://github.com/morris-lab/Colony-counter.

#### Quantitative PCR and analysis

Cells were collected for RNA extraction (RNeasy kit; Qiagen) on day 12 of reprogramming and RT using the Maxima RT kit (Thermo Fisher Scientific, K1672). A total of 20 ng of RT RNA was mixed with TaqMan Gene Expression Master Mix (Thermo Fisher Scientific) and gene-specific TaqMan probes (Supplementary Table 11) in a 20 µl reaction volume and processed according to manufacturer’s instructions (4371135) on the StepOne Plus qPCR system. Per gene fold change for Foxd2 overexpressing cells was calculated relative to control reprogramming cells (*Hnf4α*–*Foxa1* and GFP control OE) that were processed in parallel, after normalization to the housekeeping gene, *Actb*.

#### Reprogramming with activin/nodal/TGF-β signaling inhibition

Cells were reprogrammed as previously described. They were cultured in hepatic media with 2.6 µM SB431542 (STEMCELL, 72232) from day 0, changing the media every 2 d. On day 5, cells were collected for qPCR analysis and processed accordingly. Additionally, colony formation assays were conducted following the procedure described above.

### Reporting summary

Further information on research design is available in the Nature Portfolio Reporting Summary linked to this article.

## Online content

Any methods, additional references, Nature Portfolio reporting summaries, source data, extended data, supplementary information, acknowledgements, peer review information; details of author contributions and competing interests; and statements of data and code availability are available at 10.1038/s41587-023-01931-4.

### Supplementary information

Supplementary Information Supplementary methods.

Reporting Summary

Supplementary Table 1 List of oligos used in the manuscript.

Supplementary Table 2 CellTag-multi allowlist.

Supplementary Table 3 Lists of lineage-specific gene expression and TF activity markers from the in vitro hematopoiesis state–fate analysis (Mann–Whitney–Wilcoxon test, two-sided; adjusted *P* values represent Benjamini–Hochberg corrected *P* values).

Supplementary Table 4 Gene Ontology results for lineage-specific gene expression markers from the in vitro hematopoiesis state–fate analysis (Fisher’s exact test, one-sided; FDR values represent Benjamini–Hochberg corrected *P* values).

Supplementary Table 5 Lists of day 3 on-target and off-target fate marker genes increasing in expression over time along respective trajectories.

Supplementary Table 6 Lists of genes differentially expression in uninduced MEFs, day 3 on-target destined and day 3 off-target destined cells (Mann–Whitney–Wilcoxon test, two-sided; adjusted *P* values represent Bonferroni corrected *P* values).

Supplementary Table 7 Lists of differentially accessible regions (DARs) in uninduced MEFs, day 3 on-target destined and day 3 off-target destined cells. Lists of on-target and off-target DARs linked to genes (Mann–Whitney–Wilcoxon test; two-sided; FDR values represent Benjamini–Hochberg corrected *P* values).

Supplementary Table 8 Lists of TF activity markers in uninduced MEFs, day 3 on-target destined and day 3 off-target destined cells.

Supplementary Table 9 List of genes differentially expressed between stalled and nonstalled populations (Mann–Whitney–Wilcoxon test, two-sided; adjusted *P* values represent Bonferroni corrected *P* values). GO analysis results for genes enriched in the stalled population of cells (Fisher’s exact test, one-sided; FDR values represent Benjamini–Hochberg corrected *P* values).

Supplementary Table 10 Lists of top 20 genes and their scores in the BMP and activin/nodal/TGF spectra factors.

Supplementary Table 11 List of TaqMan probes used in this manuscript.

Supplementary Table 12 List of exact *P* values of Figs. 4e and 5f, and Extended Data Figs. 5c,d and 10f.

## Data Availability

Data associated with this work is available at GEO accession GSE216521 (ref. ^82^). Pooled CellTag-multi libraries have been deposited at Addgene: https://www.addgene.org/pooled-library/morris-lab-celltag (pSMAL-CellTag-multi-v1 barcode library #206045).

## Code availability

Software and detailed documentation for CellTag and clonal analysis are available at https://github.com/morris-lab/newCloneCalling. Processed single-cell objects and custom code to reproduce analyses and figures is available at https://github.com/morris-lab/CellTag-multi-2023.
