# Supplementary material for: Single-cell lineage capture across genomic modalities with CellTag-multi reveals fate-specific gene regulatory changes
Source: Nat Biotechnol. 2023 Sep 25;42(6):946–59. doi: 10.1038/s41587-023-01931-4 (PMC11180607; doi:10.1038/s41587-023-01931-4)
Supplement: Supplementary file 1 — Supplementary methods. [file 41587_2023_1931_MOESM1_ESM.pdf]

nature biotechnology

Article

https://doi.org/10.1038/s41587-023-01931-4

Single-cell lineage capture across genomic modalities with CellTag-multi reveals fate-specific gene regulatory changes

In the format provided by the authors and unedited

Supplementary Experimental Methods

CellTag amplification for scRNA-seq. Targeted PCR was used to amplify CellTag barcodes from the single-cell cDNA library, obtained after step 2.4 of the 10x Genomics 3' scRNA user guide (CG000315). 5ul (or at least 60ng) of cDNA was mixed with 2x Q5 HF PCR Master Mix (New England Biolabs) and 500nM of *P5/R1-par* and *P7/SI-R2* primers in a 50ul reaction volume and subjected to the following PCR program: 98 C for 30s; N cycles (98ºC for 10s; 54ºC for 30s; 72ºC for 30s); 72ºC for 2 minutes. The number of PCR cycles (N) was kept the same as the number of cycles used during sample index PCR of the main scRNA-seq library. CellTag amplicons was purified using double-sided bead purification (0.4x-0.64x), quantified on an Agilent TapeStation and sequenced either by themselves (with a 50% Phi-X) or pooled with scRNA-seq libraries (preferred). Primer sequences are available in Supplementary Table 1.

scATAC-seq library preparation with modifications for CellTag capture. To prepare scATAC-seq libraries with CellTag capture, nuclei were isolated using manufacturer's instructions (CG000169), centrifuged to remove supernatant, and lightly fixed in 100ul 0.1% formaldehyde solution for 5 minutes. The reaction was stopped for 5 minutes by adding 30ul of stop buffer (0.625M Glycine, 0.5% BSA, 0.25M ph8 Tris-Cl in PBS). The nuclei suspension was diluted using 100ul diluted nuclei buffer (10x Genomics; CG000169) and pelleted using centrifugation. The pellet was subjected to tagmentation for 60 minutes after re-suspension in a 15ul tagmentation reaction (for up to 15k nuclei) according to the manufacturer's instructions (CG000209). After tagmentation, the reaction mixture was diluted with 100ul dilute nuclei buffer, nuclei were pelleted using centrifugation and subjected to targeted in situ reverse transcription in a 100ul reaction volume (20ul of 5x SuperScript IV reaction buffer, 5ul each of dNTPs, DTT, RnaseOUT RNase inhibitor, SuperScript IV Reverse Transcriptase, 1uM of primer *ctac2-rt1*) using the following temperature program: 4ºC for 2 minutes; 10ºC for 2 minutes; 20ºC for 2 minutes; 30ºC for 2 minutes; 40ºC for 2 minutes; 45ºC for 10 minutes. After isRT, 100ul dilute nuclei buffer was added and nuclei were pelleted using centrifugation. 15ul GEM-nuclei mix was prepared to load nuclei on 10x Genomics Chip E/H by mixing up to 15k nuclei with 6ul of ATAC buffer (10x Genomics: 2000193) and 3ul of 4uM primer *ctac2-rt1*. Any remaining volume was made up with dilute nuclei buffer. GEM-nuclei mix was loaded onto Chip E/H along with ATAC GEM beads and barcoding enzyme mix, the remaining steps of the scATAC-seq library preparation protocol were performed according to the manufacturer's instructions. Primer sequences are available in Supplementary Table 1. All centrifugation steps were performed at 500g for 10 minutes at 4ºC unless stated otherwise.

CellTag amplification from scATAC-seq. While CellTags can be recovered directly from the sequenced scATAC-seq library, a higher yield can be obtained using an additional targeted PCR step. For this, 5ul of the library is collected after step 3.2 of the user guide (CG000209) and mixed with 2x Q5 HF master mix, 500nM of primer *biot-atac2_lin* and water in a 50ul reaction volume, and CellTag containing fragments are linearly amplified using the following PCR program: 98ºC for 30s; 20 cycles (98ºC for 10s; 67ºC for 30s; 72ºC for 30s); 72ºC for 2 minutes. The CellTag amplicons are purified using streptavidin-coated magnetic bead pulldown (ThermoFisher Scientific 65001) and purified fragments are resuspended in 20ul of water. A final sample index PCR is performed to create a sequencible library in presence of 2x Q5 master mix, 500nM each of *partial_p5* and *biot-atac2_e-rev* primers in a 100ul reaction volume using the following PCR program: 98ºC for 30s; 13 cycles (98ºC for 10s; 67ºC for 30s; 72ºC for 30s); 72ºC for 2 minutes and libraries are purified using a double-sided bead cleanup, as described in Step 4.2 of 10x Genomic scATAC-seq user guide (CG000209). Primer sequences are available in Supplementary Table 1.

CellTag-multi library synthesis. CellTag-multi library was synthesized using Restriction Free (RF) cloning[1]. CellTag-multi barcodes were obtained as a gBlock from IDT DNA (see Supplementary Table 1 for sequence) and cloned into the pSMAL-ctac2 vector. 20ng of the CellTag-multi-v1 gBlock and 100ng of pSMAL-ctac2 vector were mixed with 2x Phusion PCR master mix in a 20ul reaction volume. The reaction mixture was subjected to the following thermal cycling program: 98ºC for 30s; 15 cycles (98ºC for 8s, 60ºC for 20s, and 72ºC for 4.5 minutes); 72ºC for 5 minutes. The parental plasmid was digested by adding 2ul of methylation-sensitive restriction enzyme, *DpnI* (New England Biolabs), and incubating the reaction at 37ºC for 2 hours followed by inactivation at 80ºC for 20 minutes. 10ul of the reaction mix was transformed directly into 100ul of Stellar chemically competent cells (Takara Bio), cells were allowed to recover at 37ºC, 250rpm in 1ml of SOC media and plated on a Nunc Square BioAssay plate (Cat. 166508). Plates were incubated overnight at 37ºC. Bacterial colonies were collected using a scraper and allowed to recover in 150ml of LB media supplemented with 100ug/ml Ampicillin. CellTag-multi libraries were purified using a Qiagen High speed maxi prep kit (Cat. 12662) and library complexity was assessed as described below. This cloning was performed four times and libraries from each round were pooled to obtain the final high complexity library.

Supplementary Computational methods

Identifying clones. Clone identification was performed based on our previously described method[2], [3]. Reads matching the CellTag-multi barcode sequence pattern $(N)_3GT(N)_3CT(N)_3AG(N)_3TG(N)_3CA(N)_3$ were extracted from single-cell bam files as obtained from CellRanger, filtered to remove false positive transcriptomic/genomic reads and reads originating from non-cell droplets. For scRNA-seq, cell barcode-CellTag-UMI triplets represented by only a single read were discarded. CellTag sequencing saturation was calculated as the percent of non-unique CellTag reads. CellTags were error-corrected using Starcode[4] to mitigate PCR/sequencing errors and filtered to remove sequences outside of the allowlist. Cell x CellTag read count (ATAC)/ UMI count (RNA) matrices were obtained, binarized and cells with too few or too many tags were removed to obtain the final Cell x CellTag matrices for scRNA-seq and scATAC-seq assays. Cell-cell similarity was computed using the Jaccard similarity metric and clones were identified using graph clustering. For clone calling across modalities, scRNA-seq and scATAC-seq CellTag matrices were merged before the Jaccard similarity calculation step. Code for clone calling can be found at: https://github.com/morris-lab/newCloneCalling

Estimating homoplasy in a lineage tracing experiment. To estimate rate of occurrence of homoplasy (i.e. unrelated cells being labelled with the same CellTag barcodes), we have developed a simple simulator function *find_homoplasy*. This is available on our GitHub repository at: https://github.com/morris-lab/newCloneCalling/.

For each cell i, the simulator assigns N_i transduction events where N_i ~ Poisson(MOI). Next, each transduction event is assigned a unique CellTag barcode. The probability of a transduction event to be assigned a CellTag j is given by p_j where p_j is the relative abundance of CellTag j reads in the allowlisting PCR data. Next, the rate of homoplasy is calculated as the expected fraction of non-unique CellTag signatures in the starting cell population after tagging.

Clone cell embedding. For clone-cell embedding, we first obtained our single-cell data as an AnnData object and computed a cell-cell connectivity matrix based on PCA (in case of scRNA-seq) or CCA (in case of joint scRNA-seq scATAC-seq embedding). Next, we created a new AnnData object containing both cells and clones as observations. The connectivity matrix in the .obsm['connectivities'] slot was expanded to introduce clones. Then, clones were connected to their constituent cells by setting the respective entries in the expanded 'connectivities' matrix to 1. Finally, we used this clone-cell AnnData object with the expanded connectivity matrix as an input to graph embedding algorithms such as UMAP or Force Atlas.

Assessing the complexity of CellTag-multi libraries and allowlisting. A list of allowed CellTag sequences for each CellTag library was created using amplicon sequencing. 50ng of CellTag plasmid library was mixed with 2x Q5 HF Master Mix, 2.5ul each of 0.5uM primers *bATAC_fwd* and *bATAC_rev* in a 25ul reaction volume and subjected to the following PCR program: 98ºC for 30s; 10 cycles (98ºC for 10s; 63ºC for 30s; 72ºC for 1 minute). Two amplicon libraries were generated from each CellTag library plasmid preparation in parallel and sequenced on an Illumina Miseq. For each replicate, reads matching the CellTag sequence pattern $(N)_3GT(N)_3CT(N)_3AG(N)_3TG(N)_3CA(N)_3$ were extracted, sequencing/PCR errors were corrected by collapsing tags within 4 edits of each other using starcode[4] and thresholded to retain CellTags containing at least N reads where N = max(10, 90th percentile/10). An allowlist was created by collecting all CellTag sequences retained in thresholded lists from both replicates. Allowlists from four separate CellTag libraries were combined to create the master allowlist for the CellTag-multi library (Supplementary Table 2). The detailed analysis code can be found at: https://github.com/morris-lab/newCloneCalling

[1] S. R. Bond and C. C. Naus, "RF-Cloning.org: an online tool for the design of restriction-free cloning projects," *Nucleic Acids Research*, vol. 40, no. Web Server issue, p. W209, Jul. 2012, doi: 10.1093/NAR/GKS396.

[2] W. Kong, B. A. Biddy, K. Kamimoto, J. M. Amrute, E. G. Butka, and S. A. Morris, "CellTagging: combinatorial indexing to simultaneously map lineage and identity at single-cell resolution," *Nat Protoc*, vol. 15, no. 3, pp. 750–772, Mar. 2020, doi: 10.1038/s41596-019-0247-2.

[3] B. A. Biddy *et al.*, "Single-cell mapping of lineage and identity in direct reprogramming," *Nature*, vol. 564, no. 7735, pp. 219–224, 2018, doi: 10.1038/s41586-018-0744-4.

[4] E. Zorita, P. Cuscó, and G. J. Filion, "Starcode: sequence clustering based on all-pairs search," *Bioinformatics*, vol. 31, no. 12, pp. 1913–1919, Jun. 2015, doi: 10.1093/BIOINFORMATICS/BTV053.
