# Supplementary material for: Single-cell lineage capture across genomic modalities with CellTag-multi reveals fate-specific gene regulatory changes
Source: Nat Biotechnol. 2023 Sep 25;42(6):946–59. doi: 10.1038/s41587-023-01931-4 (PMC11180607; doi:10.1038/s41587-023-01931-4)
Supplement: Supplementary file 2 — Reporting Summary [file 41587_2023_1931_MOESM2_ESM.pdf]

nature portfolio

Corresponding author(s): Samantha A Morris

Last updated by author(s): Mar 12, 2023

Reporting Summary

Nature Portfolio wishes to improve the reproducibility of the work that we publish. This form provides structure for consistency and transparency in reporting. For further information on Nature Portfolio policies, see our Editorial Policies and the Editorial Policy Checklist.

Statistics

For all statistical analyses, confirm that the following items are present in the figure legend, table legend, main text, or Methods section.

n/a	Confirmed	
☐	☒	The exact sample size (*n*) for each experimental group/condition, given as a discrete number and unit of measurement
☐	☒	A statement on whether measurements were taken from distinct samples or whether the same sample was measured repeatedly
☐	☒	The statistical test(s) used AND whether they are one- or two-sided<br>*Only common tests should be described solely by name; describe more complex techniques in the Methods section.*
☒	☐	A description of all covariates tested
☐	☒	A description of any assumptions or corrections, such as tests of normality and adjustment for multiple comparisons
☐	☒	A full description of the statistical parameters including central tendency (e.g. means) or other basic estimates (e.g. regression coefficient) AND variation (e.g. standard deviation) or associated estimates of uncertainty (e.g. confidence intervals)
☐	☒	For null hypothesis testing, the test statistic (e.g. *F*, *t*, *r*) with confidence intervals, effect sizes, degrees of freedom and *P* value noted<br>*Give P values as exact values whenever suitable.*
☒	☐	For Bayesian analysis, information on the choice of priors and Markov chain Monte Carlo settings
☒	☐	For hierarchical and complex designs, identification of the appropriate level for tests and full reporting of outcomes
☐	☒	Estimates of effect sizes (e.g. Cohen's *d*, Pearson's *r*), indicating how they were calculated

Our web collection on statistics for biologists contains articles on many of the points above.

Software and code

Policy information about availability of computer code

Data collection	Cellranger (https://support.10xgenomics.com/single-cell-gene-expression/software/downloads/latest), Cellranger-atac (https://support.10xgenomics.com/single-cell-atac/software/downloads/latest) and Cellranger-ARC (https://support.10xgenomics.com/single-cell-multiome-atac-gex/software/downloads/latest) were used to process data collected using the 10x Chromium 3' scRNA, 10x scATAC and 10x multiome platforms respectively.
Data analysis	1. Seurat v4 (https://satijalab.org/seurat/)<br>2. Scanpy 1.9.1 (https://scanpy.readthedocs.io/en/stable/)<br>3. ArchR v1.0.1/v2 (https://www.archrproject.com/)<br>4. HOMER v2 (http://homer.ucsd.edu/homer/motif/)<br>5. sci-kit learn 1.0 (https://scikit-learn.org/stable/)<br>6. SHAP 0.40.0 (https://shap.readthedocs.io/en/latest/)<br>7. CellTag analysis code (https://github.com/morris-lab/newCloneCalling)<br>8. Python v3 (https://www.python.org/download/releases/3.0/)<br>9. FigR 1.0.1 (https://github.com/buenrostrolab/FigR)<br>10. Spectra (https://github.com/dpeerlab/spectra)<br>11. Capybara (https://github.com/morris-lab/Capybara)<br>12. CoSpar v0.3.0 (https://github.com/AllonKleinLab/cospar)<br>13. Meme-chip 5.5.3 (https://meme-suite.org/meme/doc/meme-chip.html)

For manuscripts utilizing custom algorithms or software that are central to the research but not yet described in published literature, software must be made available to editors and reviewers. We strongly encourage code deposition in a community repository (e.g. GitHub). See the Nature Portfolio guidelines for submitting code & software for further information.

Data

Policy information about availability of data

All manuscripts must include a data availability statement. This statement should provide the following information, where applicable:

- Accession codes, unique identifiers, or web links for publicly available datasets
- A description of any restrictions on data availability
- For clinical datasets or third party data, please ensure that the statement adheres to our policy

Data associated with this work is available at GEO accession GSE216521;
mm10 reference genome (https://support.10xgenomics.com/single-cell-gene-expression/software/downloads/latest);
ENCODE cCRE database (https://screen.encodeproject.org/);

Human research participants

Policy information about studies involving human research participants and Sex and Gender in Research.

Reporting on sex and gender	N/A
Population characteristics	N/A
Recruitment	N/A
Ethics oversight	N/A

Note that full information on the approval of the study protocol must also be provided in the manuscript.

Field-specific reporting

Please select the one below that is the best fit for your research. If you are not sure, read the appropriate sections before making your selection.

☒ Life sciences ☐ Behavioural & social sciences ☐ Ecological, evolutionary & environmental sciences

For a reference copy of the document with all sections, see nature.com/documents/nr-reporting-summary-flat.pdf

Life sciences study design

All studies must disclose on these points even when the disclosure is negative.

Sample size	Sample size was not determined a priori. For single-cell experiments, comparisons were performed between groups of cells large enough to sufficiently power the analyses. For other experiments, a minimum of 3 independent biological replicates were performed in case significant statistical results were inferred.
Data exclusions	For single cell experiments, low quality cell barcodes were filtered based on standard metrics in the field (for RNA: percent mitochondrial reads, number of UMIs and number of genes per cell; for ATAC: TSS enrichment scores, number of unique fragments per cell). No data was excluded from analysis.
Replication	Two Independent biological replicates were used for direct reprogramming analysis to determine reproducibility, both of which were successful; two and six biological replicates were performed for Foxd2 and Zfp281 colony formation assays respectively. All replicates were successful and have been included in the study.
Randomization	There were no variables to randomize in this study.
Blinding	Blinding was not relevant to single cell time courses as only a single condition was being tested. Blinding was performed for qPCR and Colony formation assays. Data processing and filtering were kept consistent across conditions within the same experiment to prevent any bias.

Reporting for specific materials, systems and methods

We require information from authors about some types of materials, experimental systems and methods used in many studies. Here, indicate whether each material, system or method listed is relevant to your study. If you are not sure if a list item applies to your research, read the appropriate section before selecting a response.

Materials & experimental systems

n/a	Involved in the study	
☐	☒	Antibodies
☐	☒	Eukaryotic cell lines
☒	☐	Palaeontology and archaeology
☐	☒	Animals and other organisms
☒	☐	Clinical data
☒	☐	Dual use research of concern

Methods

n/a	Involved in the study	
☒	☐	ChIP-seq
☒	☐	Flow cytometry
☒	☐	MRI-based neuroimaging

Antibodies

Antibodies used	E-Cadherin; BD Biosciences; Cat 610182; Clone 36/E
Validation	This is a broadly-used and validated antibody, as detailed here: https://www.bdbiosciences.com/en-us/products/reagents/microscopy-imaging-reagents/immunofluorescence-reagents/purified-mouse-anti-e-cadherin.610182

Eukaryotic cell lines

Policy information about cell lines and Sex and Gender in Research

Cell line source(s)	293T-17; ATCC: CRL-11268
Authentication	ATCC performed STR profiling following ISO 9001 and ISO/IEC 17025 quality standards. No additional authnetication was performed on our end.
Mycoplasma contamination	Cell lines are tested for mycoplasma contamination routinely. Results are consistently negative.
Commonly misidentified lines (See ICLAC register)	No commonly misidentified lines were used in this study.

Animals and other research organisms

Policy information about studies involving animals; ARRIVE guidelines recommended for reporting animal research, and Sex and Gender in Research

Laboratory animals	Mouse strain C57BL/6J was used for this study. E13.5 embryos were used for isolating embryonic fibroblasts and 8 week old adult mice were used for isolating hematopoietic progenitor cells.
Wild animals	No wild animals were used for this study.
Reporting on sex	Sex was not considered in study design.
Field-collected samples	No field samples were collected for this study.
Ethics oversight	Institutional Animal care and Use Committees at Washington University in St Louis.

Note that full information on the approval of the study protocol must also be provided in the manuscript.
